# Population diversification in the frog *Mantidactylus bellyi* on an isolated massif in northern Madagascar based on genetic, morphological, bioacoustic and ecological evidence

**DOI:** 10.1371/journal.pone.0263764

**Published:** 2022-03-31

**Authors:** Safidy M. Rasolonjatovo, Mark D. Scherz, Robin Schmidt, Julian Glos, Andolalao Rakotoarison, Achille P. Raselimanana, Miguel Vences

**Affiliations:** 1 Mention Zoologie et Biodiversité Animale, Université d’Antananarivo, Antananarivo, Madagascar; 2 Association Vahatra, Lot V A 38 LBA Ter Ambohidempona Tsiadana, Antananarivo, Madagascar; 3 Division of Evolutionary Biology, Zoologisches Institut, Technische Universität Braunschweig, Braunschweig, Germany; 4 Natural History Museum of Denmark, University of Copenhagen, Copenhagen Ø, Denmark; 5 Institute of Zoology, Animal Ecology and Conservation, Universität Hamburg, Hamburg, Germany; National Taiwan Normal University, TAIWAN

## Abstract

In the processes that give rise to new species, changes first occur at the population level. But with the continuous nature of the divergence process, change in biological properties delimiting the shift from “individuals of divergent populations” towards “individuals of distinct species”, as well as abiotic factors driving the change, remain largely ambivalent. Here we study diversification processes at the population level in a semi-aquatic frog, *Mantidactylus* (*Brygoomantis*) *bellyi*, across the diverse vegetation types of Montagne d’Ambre National Park (MANP), Madagascar. Genetic diversity was assessed with seven newly developed microsatellite markers as well as mitochondrial DNA sequences and concordance with patterns of ecological, morphological, and bioacoustic divergence evaluated. We found *M*. *bellyi* lacking mitochondrial differentiation within MANP, while microsatellite datasets partitioned them into three highly differentiated, geographically separated subpopulations (with indications for up to five subpopulations). The molecular grouping–primarily clustering individuals by geographic proximity–was coincident with differences in mean depth and width of waters, suggesting a possible role of fluvial characteristics in genetic exchange in this stream-breeding species. Genetic clustering not consistent with differences in call properties, except for dominant call frequencies under the two-subpopulations model. Morphological divergence was mostly consistent with the genetic clustering; subpopulations strongly differed by their snout-vent length, with individuals from high-elevation subpopulations smaller than those from populations below 1000 m above sea level. These results exemplify how mountains and environmental conditions might primarily shape genetic and morphological divergence in frog populations, without strongly affecting their calls.

## Introduction

Delimitation of species boundaries is a controversial topic in biodiversity research. To alleviate ambiguity, species limits are currently preferentially assessed through integrative approaches where molecular analyses play a critical role [1, 2]. In anuran amphibians, species are ideally delimited based on congruent results from bioacoustic, morphological and molecular analyses, and with a special focus on assessing gene flow at contact zones [3–6]. Understanding that under every species concept, species are independent evolutionary lineages [7] has been a major advance, yet how to operationally delimit lineages that are independent enough to be considered as species remains disputed. For instance, Sukumaran & Knowles [8] suggested that commonly used species delimitation approaches based on Bayesian inference of multispecies coalescence delimit population-level structure, which often does not correspond to distinct species.

Tightly linked to species delimitation is the study of the process of species formation. It is generally assumed that allopatric processes, i.e., the inhibition of gene flow due to geographical barriers, is the prevalent mechanism by which new species arise [9]. On the other hand, the importance of adaptation in generating initial divergences of groups of individuals has been shown in multiple case studies, but what proportion of such ecogeographically isolated populations evolve into distinct species remain an important unresolved issue [10, 11]. In general, independent from the geography of speciation, i.e., allopatric vs. sympatric, the importance of ecological adaptation and differentiation across ecotones has been identified as an important field of research [12, 13].

As a promising model region for studying species diversification [14, 15], Madagascar attracts substantial attention from researchers working in evolution. This is due to its long period of isolation from other land masses, which has allowed its biota to evolve independently, its locally diversified ecoregions and heterogeneous ecosystems driving high rates of microendemism and numerous evolutionary radiations. Presumably, mountain massifs in the north of the island favor adaptive speciation due to their considerable elevational range and their topographic location [14, 16]. We focused the present study on one of these massifs, the Montagne d’Ambre. This massif is protected as Montagne d’Ambre National park (MANP) and contains several different vegetation types, mainly being dominated by a medium elevation moist evergreen forest [17]. Previously connected to other forest blocks, this massif is now separated from the large block of eastern rainforest to its south by drier lowland habitat [18–21]. Populations housed in such a montane refugium would remain isolated from the surrounding habitats and isolation between populations could be sharpened by the substantial habitat differences existing on MANP, driven by gradual change of bioclimatic conditions over the massif’s elevational profile [22–24].

Studies on population genetics of Malagasy fauna have generally been focused on animals with high dispersal abilities like lemurs [25, 26], bats [27–29], and birds [30], or on parasite-host and vectors of human diseases [31–34]. An enormous amount of genetic research has also focused on amphibians in Madagascar. Although the majority of these focused on DNA barcoding, phylogenetics and species delimitation, several in-depth studies have assessed the population-level genetic variation of amphibian species (e.g., [35–42]). Because amphibians–especially small-sized species–have a limited dispersal capacity compared to other groups of terrestrial vertebrates, and due to their usually biphasic lifestyle often having specific requirements of both aquatic and terrestrial habitats, they constitute a suitable model group for studies on the comparative influence of adaptive versus non-adaptive processes of species formation [43].

Mantellid frogs represent the most species-rich clade of the Malagasy amphibians [44]. Considerable taxonomic revisions have targeted this family (e.g., [45–48]) though some mantellid genera remain challenging for taxonomists. Among these is the genus *Mantidactylus* where taxonomic revisions were made difficult by the low-intensity, irregular calls of several species, polymorphism, noticeable intraspecific divergence despite morphological similarities, and existence of synonymies and uncertainties in their original descriptions [44]. This genus is one of the least studied group in Madagascar, with a limited number of recent findings in terms of taxonomy [48–52], natural history [53–56], diversity of chemical communication [57, 58], phylogeography [4], biology [59, 60] and physiology [61].

Our study species, *Mantidactylus* (*Brygoomantis*) *bellyi*, is a semi-aquatic frog present across northern Madagascar and distributed across the entire elevational gradient of Montagne d’Ambre. We here aim to investigate whether gene flow might be reduced among populations of *M*. *bellyi* exposed to different ecological conditions on MANP, and whether possible genetic divergences between populations would be reflected by morphological and bioacoustic divergences. We assessed fine-scale genetic variation within *M*. *bellyi* populations across MANP with newly developed microsatellite markers, and subsequently tested for ecological, morphological, and bioacoustic differences between subpopulation clusters revealed by the genetic data. Our multifaceted study sheds light on population-level differentiation processes in one of the least studied genera of Malagasy frogs and informs our understanding of population-boundaries in tropical montane amphibians.

## Materials and methods

### Study site

**Fig 1:**
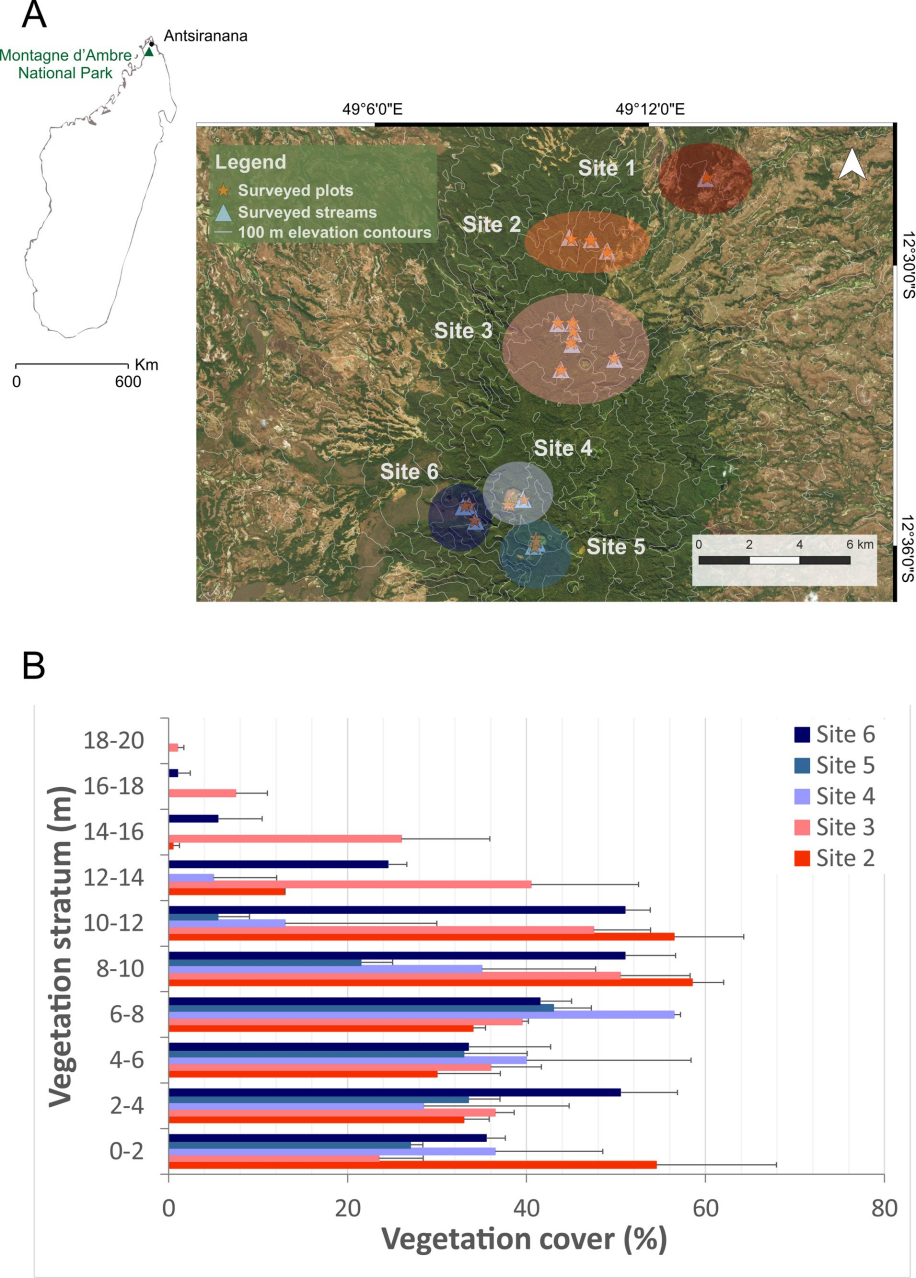
Habitat survey sites and their respective vegetation cover intervals. A] Map showing the location of Montagne d’Ambre National Park and the different collection localities of ecological variables within the park are represented by orange stars for plots and light blue triangles for streams. The localities were grouped *a priori* into six sites based on geographical proximity as emphasized by the differently colored large circles, which also range from dark red at lowest elevations to dark blue at higher elevations, except the site on the western slope, which is colored in navy blue. B] Vegetation cover at sites 2–6 obtained from the two replicates of a 100-m-long linear transect at each site. Satellite imagery of the park were taken from Microsoft® Bing™ Maps.

Fieldwork was carried out in Montagne d’Ambre National Park located between 12.34–12.75° S and 49.05–49.26° E [24], in the extreme north of Madagascar under fieldwork permit N°19117-MEEF/SG/ DGF/DSAP/SCB.Re). During the rainy season of November 2017 –January 2018, streams, waterfalls, lakes and pools were surveyed for the presence of *Mantidactylus* (*Brygoomantis*) *bellyi*. They were selected to cover different elevations (from 467 to 1394 m above sea level) and diversified forest structures within the massif. The 18 collection localities were subsequently *a priori* grouped into six groups, here called sites 1–6, based on geographical proximity (Fig 1A).

### Ecological sampling

To get an estimation of the vegetation cover per site in MANP, two replicates of a 100 m linear transect [62] were created per site, except at site 1 due to shortage of time in the field and the cyclone (‘Ava’) that hit the area in January 2018. Each linear transect within one pair was separated by ca. 20 m. Along each transect, we recorded the height of contacts with the vegetation at two-meter intervals, using a vertical pole (up to a height of six meters) and estimated it visually (> six meters). We counted all contacts between the vegetation and the pole (and its imaginary extension) at each two-meter-height interval until reaching the canopy (approximatively 10–16 m height, with emergent trees sometimes reaching 20 m height). We then used the mean from the two transect replicates to calculate the vegetation cover per interval of height, expressed as a percentage. A distinct layer was defined when the variation in vegetation cover was greater than 10% between two successive intervals of height. This dataset also enables the establishment of the schematic profile of the vegetation [63], but was not included in the statistical analyses.

To characterize the habitat of *M*. *bellyi*, we identified two categories of environmental variables: the “forest habitat” (forest areas surrounding streams, lakes, and rapids) and the “breeding sites” (including streams and rapids, but discarding variables from lentic waters, such as lakes and ponds, to ensure comparability between water variables). In the rainforest surrounding the breeding streams, we randomly selected two 5x5 m quadrats on each side of the surveyed breeding streams. Within each quadrat, we recorded the canopy cover with a spherical densiometer and took four readings per quadrat facing North, East, South and West. We counted the number of unfilled canopy opening squares. Each measurement was multiplied by 4.17 [64, 65], then subtracted from 100% to determine the canopy cover. For each forest variable, the mean values between the two surveyed quadrats were used for final analyses. To get the litter depth (mm) and the pH of the soil, we made five measurements using a graduated ruler and an Alotpower soil tester, respectively, and averaged them. The number of plants with a diameter at breast height (DBH) greater than 5 cm was counted. The height of the tallest tree (m), the height of the tree trunk (distance from the ground to the first large branch of the highest tree (m)), and the minimal and maximal diameters of tree crowns (m) of the respective tree trunks inside the quadrat were estimated by eye. The DBH of the tree with the greatest DBH (cm) was measured with a tape measure. From both quadrats, the mean value of each forest variable was calculated.

To assess physico-chemical variables of the breeding waters of *M*. *bellyi*, we randomly selected a 14–100 m straight part of the stream course (when assessing streams), or a part along the shore (for rapids). The stream slope was calculated based on the length of the stream section and the difference between its maximum and minimum elevation. Further water variables were the mean width (m), the mean depth (cm) and the mean pH of the stream (measured with a Neuftech®pH meter) by averaging ten punctual measurements along the stream section.

### Bioacoustic sampling

We searched for individuals of *Mantidactylus bellyi* using opportunistic visual surveys during the day and at night, specifically targeting suitable microhabitats and guided by the calls of the species when heard. Specimens were typically perched on rocks or half-immersed in the water, sometimes found along the edge of the stream. Call recording times were independent of time of the day since *M*. *bellyi* is a cathemeral species. Before recording the calls, body temperature of the observed male was taken with an infrared laser thermometer (Benetech® GM700–GM900, Shenzhen Jumaoyuan Science and Technology Co., Ltd.). The recording device was at a maximum distance of one meter from the observed individual, without physical contact with them. Vocalizations were recorded with a Tascam DR-05 linear PCM recorder (TEAC Europe GmbH) using the internal microphone, positioned at less than one meter from the calling males. Recordings comprised 2–27 advertisement call sequences for each male. We measured the snout–vent length of the calling specimen before release (see next paragraph).

### Morphological measurements

For each of 329 adults of *Mantidactylus bellyi* captured, sex, date and time of capture were noted. Geographic coordinates of the collection locality were registered with a Global Positioning System receiver (GPSMAP®64s GARMIN). Snout–vent length (SVL), head length (HL), maximum head width (HW), horizontal eye diameter (ED), tympanum diameter (TD), humerus length (LHU), forearm length (FOL), thigh length (THL), tibia length (TL), tarsus length (TRL) and length of the third toe (TOEL) of each captured specimen were subsequently measured using a Wiha 41102 DialMax caliper to the nearest 0.1 mm.

### Tissue sampling, DNA extraction and sequencing of the *16S* rRNA gene

All research methods reported in this paper complied with the guidelines for field research compiled by the American Society of Ichthyologists and Herpetologists (ASIH), the Herpetologists’ League (HL), and the Society for the Study of Amphibians and Reptiles (SSAR), and adhered to the legal requirements of Malagasy authorities. Approval for this study by an Institutional Animal Care and Use Committee (IACUC) was not required by Malagasy law. Approval of sampling procedures was included in the fieldwork permit issued by the Ministry of Environment, Direction of the System of Protected Areas (Fieldwork permit N°19117-MEEF/SG/ DGF/DSAP/SCB.Re). Samples were obtained by clipping a small piece of web. Individuals were treated locally with a pain relieving, antiseptic and antibacterial Bactine® spray for reducing animal suffering and disinfection. Tissue samples were stored in tubes filled with pure ethanol. Ten individuals were collected as voucher specimens to serve as reference material from across the massif. These were sedated and subsequently euthanized by immersion in MS-222 solution, in compliance with the American Veterinary Medical Association guidelines. Specimens were then fixed with 99% ethanol before transfer to 70% ethanol for long-term storage. Voucher specimens were deposited in the zoological collections of the Mention Zoologie et Biodiversité Animale of the University of Antananarivo, Madagascar (UADBA) and Zoologische Staatssammlung München, Germany (ZSM), with field numbers of M.D. Scherz (MSZC).

Total DNA of each individual was isolated using proteinase K digestion, followed by a standard salt extraction protocol [66]. To test for molecular similarity between the samples of *Mantidactylus bellyi* and to allow comparisons of a population-level differentiations within one species, we preliminarily analyzed mtDNA sequences of 136 individuals from Montagne d’Ambre National Park (MANP), and 18 specimens from other known occurrence localities of the species. A 473 bp fragment of the mitochondrial gene for *16S* rRNA was amplified in a 12.50 μl polymerase chain reaction (PCR) volume containing of 2.50 μl 5x colorless GoTaq reaction buffer, 0.10 μl GoTaq DNA polymerase (5 U/ml), 0.25 μl dNTPs (10 mM), 0.30 μl of each primer (10 μM), 8.05 μl of MQ H_2_O, and 1 μl of DNA with the following PCR conditions: 90 s at 94° C for denaturation, followed by 33 cycles of (45 s at 94° C, 45 s at 55° C, 90 s at 72° C) for amplification and a final extension of 300 s at 72° C. 16SAL (5’-CGCCTGTTTATCAAAAACAT-3’) and 16SBH-NEW (5’-CCTGGATTACTCCGGTCTGA-3’) were respectively used as forward and reverse primers. Amplified products were purified and directly sequenced on an automated ABI 3130xl DNA Genetic Analyzer.

### Development, selection and test of microsatellite primers

To assess intraspecific population-level differentiation, a microsatellite library was specifically developed for *M*. *bellyi*. For this purpose, we extracted genomic DNA from muscle tissue samples of four specimens of the species and submitted the pooled DNA to the Sequencing Genotyping Facility, Cornell Life Sciences Core Laboratory Center (CLC), USA. Here, digestion of DNA was carried out in three separate reactions with the restriction enzymes AluI, RsaI, and Hpy166II, and products were combined in equal amounts after heat inactivation of the restriction enzymes. The blunt ends were adenylated (+A) with Klenow (exo-) and dATP, and after heat inactivation of the Klenow (exo-), and reactions supplemented with ATP to 1 mM and an Illumina Y-adaptor was ligated with T4 DNA ligase. Enrichment of the fragments for microsatellites was carried out by hybridization to and magnetic capture of biotinylated repeat probes (representing two unique dimers, five unique trimers, seven unique tetramers and two unique pentamers), followed by amplification and barcoding by PCR, and sequencing on an Illumina MiSeq instrument (2 × 250 bp paired reads). Raw reads were assembled using SeqMan NGen (version 11), and the program msatcommander 1.0.8_beta (for Mac OSX) was used to scan the assembly for microsatellite loci and automatically design primer pairs. The constructed library contained 46,565 proposed microsatellite markers with minimum consecutive perfect repeat lengths of at least six (12 bp) for any dimer and at least five for any trimer, tetramer, or pentamer and PCR product size of 150–450 bp, and is available from Figshare under https://doi.org/10.6084/m9.figshare.16803556.v1. We selected from this library 30 potential loci based on the criteria (i) tetranucleotides, (ii) repeat count between 10 and 15, (iii) read count less than 1,000, (iv) GC content of roughly 50/50, (v) and pair product size not above 400 bp [67]. Primers were diluted 1:10 using MQ H_2_O and tested for PCR success with 12.50 μl reaction volume containing 2.50 μl 5x colorless GoTaq reaction buffer, 0.25 μl dNTPs (10 mM), 0.30 μl forward primer (10 μM), 0.30 μl reverse primer (10 μM), 0.10 μl GoTaq DNA polymerase, and 8.05 μl MQ H_2_O, where we added 1 μl of DNA. Amplification involved one cycle of initialization at 94° C for 300s, followed by 30 cycles of (94° C for 30 s, 60° C for 45 s, 72° C for 45 s), and a final elongation at 72° C for 600 s. Products were held at 8° C. After screening amplification success of the 30 candidate markers and estimating variation on agarose gel images, we retained 14 loci for further analyses.

### Microsatellite amplification and preparation for genotyping

For genotyping, we ran PCRs following the M13 protocol of Schuelke [68] in 12.50 μl reaction with 2.50 μl 5x colorless GoTaq reaction buffer, 0.25 μl dNTPs (10 mM), 0.30 μl of forward, 0.30 μl of reverse primer (10 μM), and 0.30 μl of FAM-, NED- or HEX-labelled primer (10 μM) 0.10 μl GoTaq DNA polymerase (5 U/ml), 7.75 μl of MQ H_2_O, and 1 μl of DNA; in a modification of the original protocol, the forward primer did not contain a M13 tail but instead a tail corresponding to an Illumina standard forward primer (5’-ACACTCTTTCCCTACACGACGCTCTTCCGATC-3’). The temperature profiles were as follows: one cycle of initialization at 94° C for 300 s, 30 cycles of (94° C for 30 s, 60° C for 45 s, 72° C for 45 s), 8 cycles of (94° C for 30 s, 53° C for 45 s, and 72° C for 45 s), with a final extension step of 72° C for 600 s before holding the products at 8° C. Before genotyping, amplified products were purified and provided with a size standard by diluting the PCR products with 15 μl HPLC H_2_O, and then mixing 15 μl GeneScan 500 ROX dye size standard with 1500 μl Hi-Di ™ formamide of which 15 μl were added to 1 μl of the diluted PCR products. This was followed by denaturation at 96° C for 5 min, then centrifugation for 2 min after stopping denaturation for 10 min on ice. Purified microsatellites were analyzed on an Applied Biosystems™ 3130xl Genetic Analyzer.

### Analysis of *16S* rRNA sequences

Chromatograms of the newly determined sequences of *16S* rRNA were quality-controlled and edited when necessary, using CodonCode Aligner v. 3.5.6 (CodonCode Corporation). Unreliable sequences were excluded. All new sequences were deposited in GenBank (accession numbers OM818671–OM818815). Genetic divergences for the *16S* gene were calculated as uncorrected pairwise distances (p-distances) in MEGA7 [69].

### Genotyping using GeneMarker

236 samples of *M*. *bellyi* were genotyped for nine microsatellite loci. Analyses of the microsatellite allele lengths and genotyping were performed in GeneMarker 1.95 (SoftGenetics, LLC) software. In general, we only kept peaks at tetrameric intervals and discarded non-reliable peaks with deviant shapes [70, 71]. In a few cases, alleles not distanced by tetrameric intervals were retained for analyses because their multiple occurrences, regular shape of peaks, and occurrence in homozygotes as well as heterozygotes suggested they are real alleles. Two of the nine loci had a high proportion of poor-quality peaks and were discarded from further analyses, reducing the final number of selected loci to seven (S1 Table in S3 File).

### Inference of genetic subpopulation clusters

We used the software STRUCTURE V.2.3.4 to infer population structure and the number of genetic clusters of individuals. The basic algorithm was described by Pritchard *et al*. [72]. Extensions to the method were published by Falush *et al*. [73, 74] and Hubisz *et al*. [75]. The implemented algorithm tries to match all individuals based on the allele frequencies of the loci so that subpopulations are in Hardy-Weinberg equilibrium and kinship relationships between subpopulations can be reconstructed [72, 76]. We performed four different models, namely the (i) model with admixture, (ii) model without admixture, (iii) model with admixture and a location prior (the collection sites; LOCPRIOR), and (iv) model without admixture and with LOCPRIOR information, using the following settings: 1–7 subpopulations (K) tested, burn-in phase of 50,000, 500,000 Markov Chain Monte Carlo (MCMC) iterations, ten repetitions of each assessment of K. Subpopulations were clustered by geographical distance between site collection (site 1 to 6). We estimated the number of subpopulations based on the graphical results of Structure combined with the statistical tests provided by Structure Harvester [77].

### Analysis of ecological variables

We ran analyses based on three datasets of environmental variables: (i) variables characterizing the breeding streams were analyzed separately, (ii) forest variables characterizing the forest areas surrounding the breeding streams were analyzed separately, (iii) stream and forest variables were analyzed together. One breeding site/stream was used as one replicate.

NMDS ordinations based on the environmental variables were performed to assess whether the breeding sites/streams and forest habitat were ecologically similar [78, 79]. NMDS statistics were calculated using the program PAST 3.26 [80], NMDS results were visualized in R 3.6.1 software [81] using the permute 0.9–5 [82], lattice Extra 0.6–28 [83] and vegan 2.5–6 [84] packages, and the stress values were obtained with the same program. We then tested if six groups/sites (defined *a priori* based on their geographical proximity) of breeding streams and forest habitat, respectively, were ecologically similar compared to other geographically distant sites by running a one-way permutational multivariate analysis of variance (perMANOVA), set at 1000 permutations. These analyses were performed in PAST 3.26, using Bray-Curtis distance measures. Finally, each environmental variable was separately compared between the sites with a Kruskal-Wallis test in R 3.6.1 [81]. These analyses were repeated to test for ecological differentiation between groups *a priori* based on the K = 2 genetic clusters and K = 3 genetic clusters.

### Analysis of acoustic signals

In total, we analyzed 230 calls from 23 males, all with associated measurements of body length and body temperature. Original audio files are available from https://doi.org/10.6084/m9.figshare.19096016.v1. Acoustic analyses were performed in Audacity 2.3.2. While visualizing the spectrogram, we first reduced the background noise using the function “equalization” in Audacity 2.3.2 by excluding acoustic signals far outside the spectral range of *M*. *bellyi* calls. When necessary, calls were also amplified using the same function. We thus retained call frequency and amplitude respectively ranging between 0–8000 Hz and -30–24 dB for analyses. Temporal call properties, namely the call duration (ms), the number of pulses (n) visually counted in the oscillogram, the resulting pulse duration (including inter-pulse interval if present) (ms), the inter-call interval (ms), and the pulse rate (n/s) were determined, as well as the dominant frequency (Hz) of each call (S1 Fig in S2 File). All temporal and spectral values were synthesized per individual based on their average.

Previous findings pointed out the inverse relationship between call frequency and body size in anurans (e.g. [85–87]), as well as the effects of environmental temperatures on temporal call traits [87]. This is especially true when body temperature (T_b_) is dependent on that of the animal’s environment such as in the case of *M*. *bellyi* [61]. Thus, the correlations between each temporal call variable and body temperature (T_b_), as well as between dominant frequency and body size (SVL), were tested with Spearman’s rank correlation tests (for non-normal data) or with Pearson’s correlation tests (for normal data) using R 3.6.1 [81]. Normality was assessed using Shapiro tests. To remove the effect of T_b_ for further analysis, we ran a regression analysis of each temporal call variable against T_b_. Dominant frequency was regressed against SVL to remove the effect of size. For each variable, the residual values were subsequently extracted and compared among sites with a Principal Component Analysis. Variation in the first two Principal Components scores and in call parameters between the sites were tested with ANOVA. A Tukey’s HSD post-hoc analysis was performed for a pairwise comparisons between sites for each call parameter based on the residuals. Graphs were built in R 3.6.1 using the ggplot2 3.2.1 [88], cowplot 1.0.0 [89], tidyverse 1.3.0 [90], dplyr 0.8.3 [91], and RColorBrewer 1.1–2 [92] R packages. The pulse rate was not included in the analyses as it is inversely correlated with pulse duration and thus would be redundant.

### Analysis of morphological variation

Morphological measurements were available for 329 adults (176 males and 153 females). As mentioned before, eleven morphological characters were taken from each individual. We created three datasets that were separately analyzed: (i) measurements of males and females pooled in one dataset, (ii) measurements of males only, and (iii) measurements of females only. All analyses were performed in R 3.6.1 [81]. After testing the normality of the data using Shapiro tests, we checked the correlation between each morphological variable and body size (or SVL) with Spearman’s rank correlation tests. To remove the effect of size, the morphometric measurements was regressed against SVL and the residual values were extracted, except for SVL for which we used the original values. The latter were subsequently compared between individuals from the six geographically distant sites with a Principal Component Analysis. Differences in morphological variables between the sites were tested with ANOVA. A Tukey’s HSD post-hoc analysis was performed for pairwise comparisons between sites for each morphological trait.

## Results

### Mitochondrial differentiation in the *16S* gene

We observed a maximum uncorrected pairwise sequence divergence of 1.3% in the mitochondrial *16S* rRNA between specimens from MANP and other occurrence localities of *Mantidactylus bellyi*, including the Ankarana Massif, and Montagne des Français. This low genetic divergence primarily confirms them as conspecific frogs, excluding the existence of multiple cryptic species hidden under the name *Mantidactylus bellyi* on MANP, as observed in the *Mantidactylus ambreensis* species complex [4, 51]. *16S* haplotype diversity in MANP was not related to genetic clusters inferred by microsatellites and is therefore not discussed further.

### Genetic diversity and population structure

**Fig 2:**
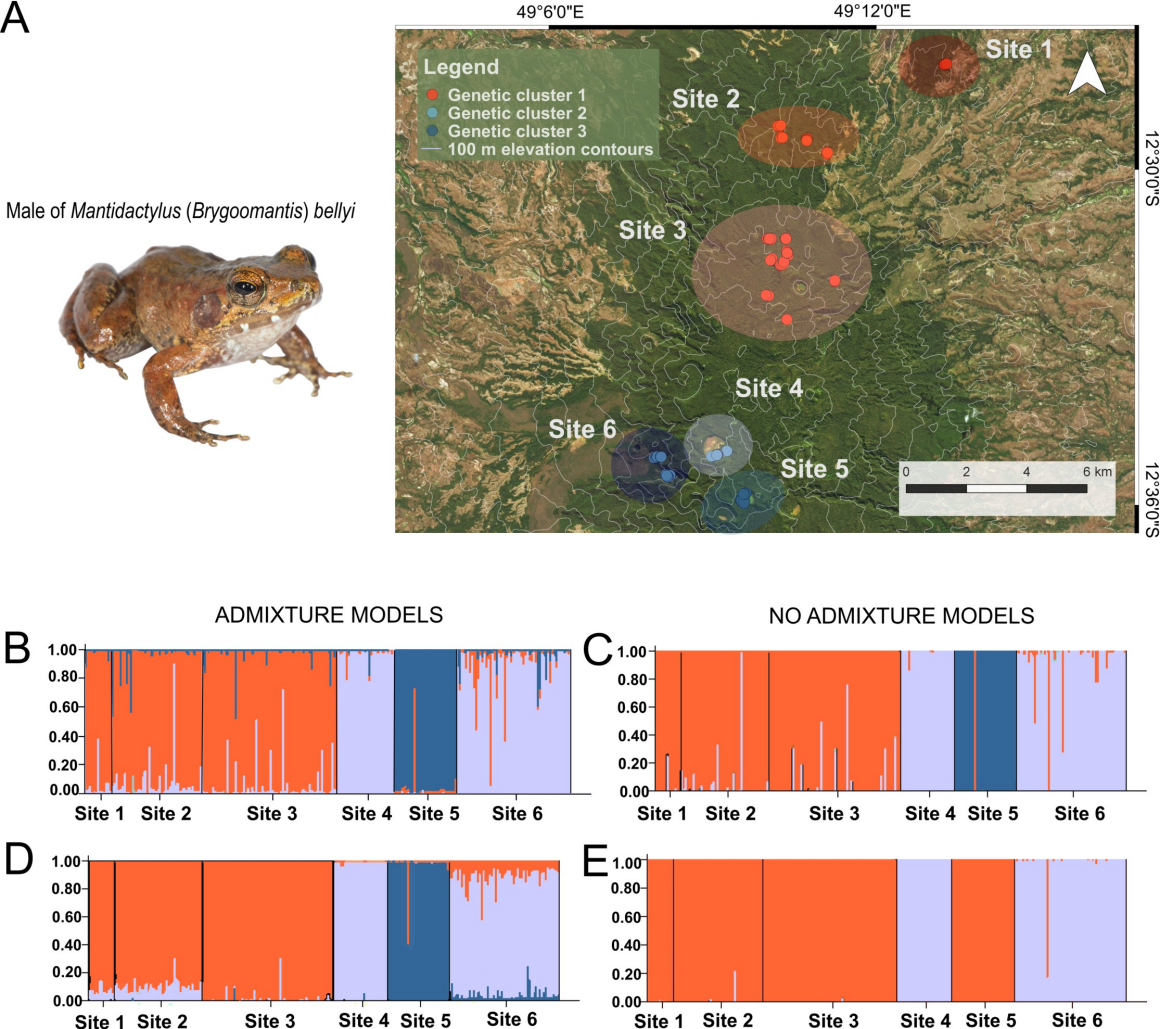
Sampled populations of *Mantidactylus bellyi* and their genetic cluster assignment. A] A male specimen of *Mantidactylus bellyi* and a map of Montagne d’Ambre, showing the *a priori* grouping into six sites based on geographical proximity as emphasized by large circles. Each dot represents a specimen of *M*. *bellyi* collected at a specific locality. The dots were colored according to the assignment of the individual to a genetic subpopulation cluster as in the K = 3 STRUCTURE models. B–E] Individual assignments of 236 individuals of *M*. *bellyi* to genetic clusters as inferred by STRUCTURE from a data set of seven microsatellites. The clustering scenarios of subpopulations K = 2–3 were depicted for the B] Admixture model (K = 3), C] No admixture model (K = 3), D] Admixture model with LOCPRIOR information (K = 3) and E] No admixture model with LOCPRIOR information (K = 2). Satellite imagery of the park were taken from Microsoft® Bing™ Maps.

The number of subpopulations for each model from the data set of seven microsatellite markers was inferred from the results of Structure Harvester given in S2 Table in S3 File. The highest ΔK was observed at K = 3 in three of the models, assuming three genetic subpopulations of *Mantidactylus bellyi* in Montagne d’Ambre National Park, whereas K = 2 was suggested by the model assuming no admixture with LOCPRIOR information (S2 Table in S3 File and Fig 2).

The plots derived from these models suggest that the genetic clusters correspond to geographically structured units (Fig 2). For models with preferred K = 3, the first cluster contained individuals from site 1, site 2 and site 3, the second cluster contained individuals living around Lac Maudit (site 4) and those inhabiting the western slope (site 6), and the third cluster contained individuals from the summit of the massif (site 5). In the no admixture / LOCPRIOR model assuming K = 2 subpopulations, individuals from site 1, site 2 and site 3 clustered together with those from site 5.

For assumed K = 4, K = 5 and K = 6 subpopulations, individuals from sites 1, 2 and 3 were grouped in the same genetic cluster, while genetic structure of individuals from site 4, site 5 and site 6 always differed from each other when considering the models with greatest ΔK (S2 Fig in S3 File). For K = 6, individuals from site 1 seemed to be genetically closer to those in site 2, compared to individuals from site 3 even though the three still formed a separate genetic cluster. This molecular pattern was half coincident with *a priori* defined sites indicating restricted gene flow between almost all sites in MANP, except between the low elevated sites 1, 2 and 3. A finest subdivision of the populations into 5 genetic clusters could be found in the model assuming an absence of admixture and with LOCPRIOR information, confirming in that case the genetic differentiation of individuals in site 3 from individuals in sites 1 and 2 (S2 Fig in S3 File).

### Structural characteristics of the sites

We identified three major vegetation layers at each sampled site, namely the herb layer, the shrub layer, and the (low and high) tree layers, with some emerging trees. Heights at which these layers occurred slightly differed between sites. Vegetation was generally denser in the tree layer (at heights > 6 m) than in the understory vegetation (Fig 1B). However, the vegetation cover of the herb layer in site 2 (55%) was almost as dense as its low tree layer (58%). Highest canopy, tallest emerging trees, as well as the minimum vegetation cover of herb layer (24%) was found at site 3 (around the “Gîte”). In contrast, emerging trees at site 5 (Grand Lac) only reached 12 m height. Lowest canopy was found at the forest areas directly surrounding the two surveyed lakes at high elevation in Montagne d’Ambre (site 4 and 5). The shrub layers, found between 2–8 m height, showed a density of 32–40% in all sites. The herb layer was mainly found between interval height of 0–2 m (Fig 1B). Total vegetation cover was 40%, 38%, 39%, 32% and 41% at site 2, site 3, site 4, site 5 and site 6.

### Ecological similarities between sites

**Fig 3:**
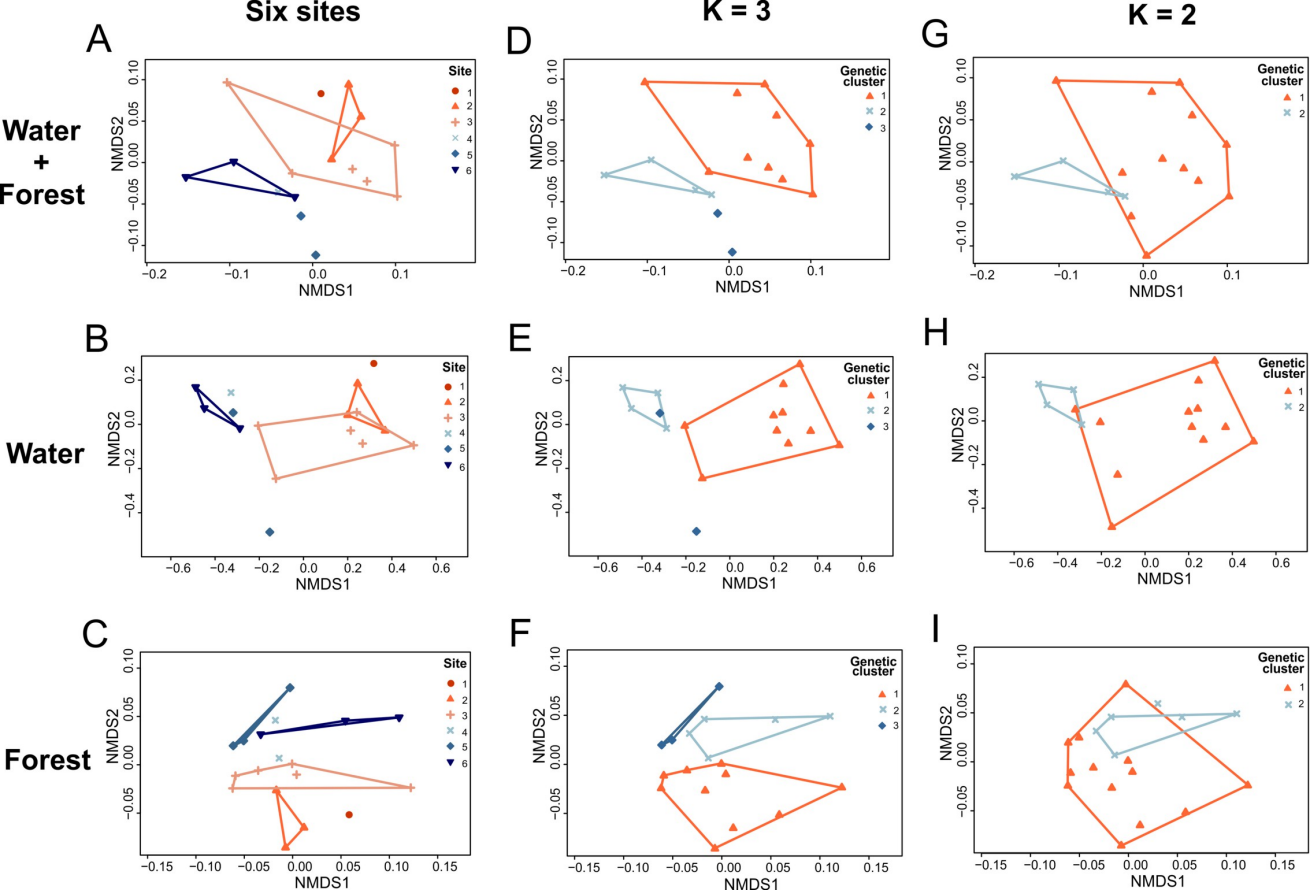
Non-metric Multidimensional Scaling (NMDS) results depicting sample points and the convex hulls for ecological datasets. Three datasets were used: variables of breeding streams of *Mantidactylus bellyi* and forest areas surrounding them analyzed together in the first row, separate analyses of the breeding streams variables in the second row, and separate analyses of the forest variables in the third row. Dots represent ecological sample points. They were colored according to their assignment to one of the six sites in the first column, to a genetic subpopulation cluster as in the K = 3 STRUCTURE models in the second column, to a genetic subpopulation cluster as in the K = 2 STRUCTURE model (No admixture/LOCPRIOR) in the third column.

The stress values of the final two-dimensional solutions of our NMDS graphs were between 0.06 and 0.15 and indicate a reasonable to good preservation of ordering relationships of the multidimensional among-transect dissimilarities (Fig 3). The six sites were not statistically different in their water and forest characteristics when analyzed together (Fig 3A, one-way perMANOVA: F = 1.73, total sum of squares = 0.29, within-group sum of squares = 0.16, P = 0.08), when analyzing only forest variables (Fig 3C, one-way perMANOVA: F = 1.15, total sum of squares = 0.27, within-group sum of squares = 0.18, P = 0.35), or when analyzing only stream variables (Fig 3B, one-way perMANOVA: F = 2.26, total sum of squares = 0.95, within-group sum of squares = 0.45, P = 0.06) although the latter analysis was close to statistical significance. No ecological variable was significantly different between the six geographically distant sites (Kruskal-Wallis test, p > 0.05 for all tests).

When testing for a grouping pattern of the ecological variables between the three genetic subpopulation clusters as in the K = 3 STRUCTURE models, we found significant evidence for grouping when stream and forest were analyzed together (Fig 3D, one-way perMANOVA: F = 2.36, total sum of squares = 0.29, within-group sum of squares = 0.22, P = 0.04) and when analyzing stream variables only (Fig 3E, one-way perMANOVA: F = 5.92, total sum of squares = 0.95, within-group sum of squares = 0.50, P < 0.01), but not when analyzing forest variables only (Fig 3F, one-way perMANOVA: F = 1.13, total sum of squares = 0.27, within-group sum of squares = 0.23, P = 0.35). A pairwise comparison revealed that ecological variables of genetic cluster 1 were significantly different from those of genetic cluster 3 (P < 0.05, Fig 3D) when stream and forest variables were analyzed together, and those of genetic cluster 1 were significantly different from those of genetic cluster 2 when analyzing only stream variables (P < 0.01, Fig 3E). All other pairwise comparisons between ecological variables of genetic clusters were not significant. Ecological variables that differed between the three genetic clusters were the mean width of waters (Kruskal-Wallis test, W = 8.61, df = 2, P < 0.05) and the mean depth of waters (Kruskal-Wallis test, W = 7.97, df = 2, P < 0.05). Among these results, we also report a near-significant difference (Kruskal-Wallis test, W = 5.96, df = 2, P = 0.05) in the maximum crown diameter of the trees between the three genetic clusters when analyzing forest variables only.

When testing for a grouping pattern of the ecological variables between two genetic clusters as in the model with no admixture and with LOCPRIOR, we found evidence for grouping when analyzing only stream variables (Fig 3H, one-way perMANOVA: F = 9.47, total sum of squares = 0.95, within-group sum of squares = 0.57, P < 0.01), but not when analyzing stream and forest variables together (Fig 3G, one-way perMANOVA: F = 1.59, total sum of squares = 0.29, within-group sum of squares = 0.26, P = 0.20), nor when analyzing only forest variables (Fig 3I, one-way perMANOVA: F = 0.52, total sum of squares = 0.27, within-group sum of squares = 0.26, P = 0.66). Ecological variables that differed between the two genetic clusters were the mean width of waters (Kruskal-Wallis test, W = 4.25, df = 1, P < 0.05) and the mean depth of waters (Kruskal-Wallis test, W = 6.4853, df = 1, P < 0.05). In general, the streams were deeper in the first three sites compared to the ones in the high-elevated site 5, with a width slowly increasing until mid-elevation (site 3) and narrowing in site 5. However, site 4 and the western site (site 6) had the shallowest and the narrowest streams, coinciding with the molecular clustering of *M*. *bellyi* inhabiting them.

### Bioacoustic variation

The advertisement calls of *Mantidactylus bellyi* consisted of a single note lasting on average 608.7 ± 277.6 ms. Therefore, in the following descriptions, call duration equals note duration (S1 Fig in S2 File). Details on call traits of *M*. *bellyi* are provided in S3 Table in S2 File. Individuals from site 5 presented the shortest calls, while the longest call durations were recorded at site 2. The species did not produce loud calls. The temporal call parameters, i.e. call duration, number of pulses, pulse duration and inter-call interval, were not significantly correlated with body temperature T_b_ (Spearman’s rank correlation test, n = 23, P > 0.05 for all tests). Dominant call frequency was inversely correlated with body size (Pearson’s correlation test, cor = -0.50, n = 23, P = 0.01).

**Fig 4:**
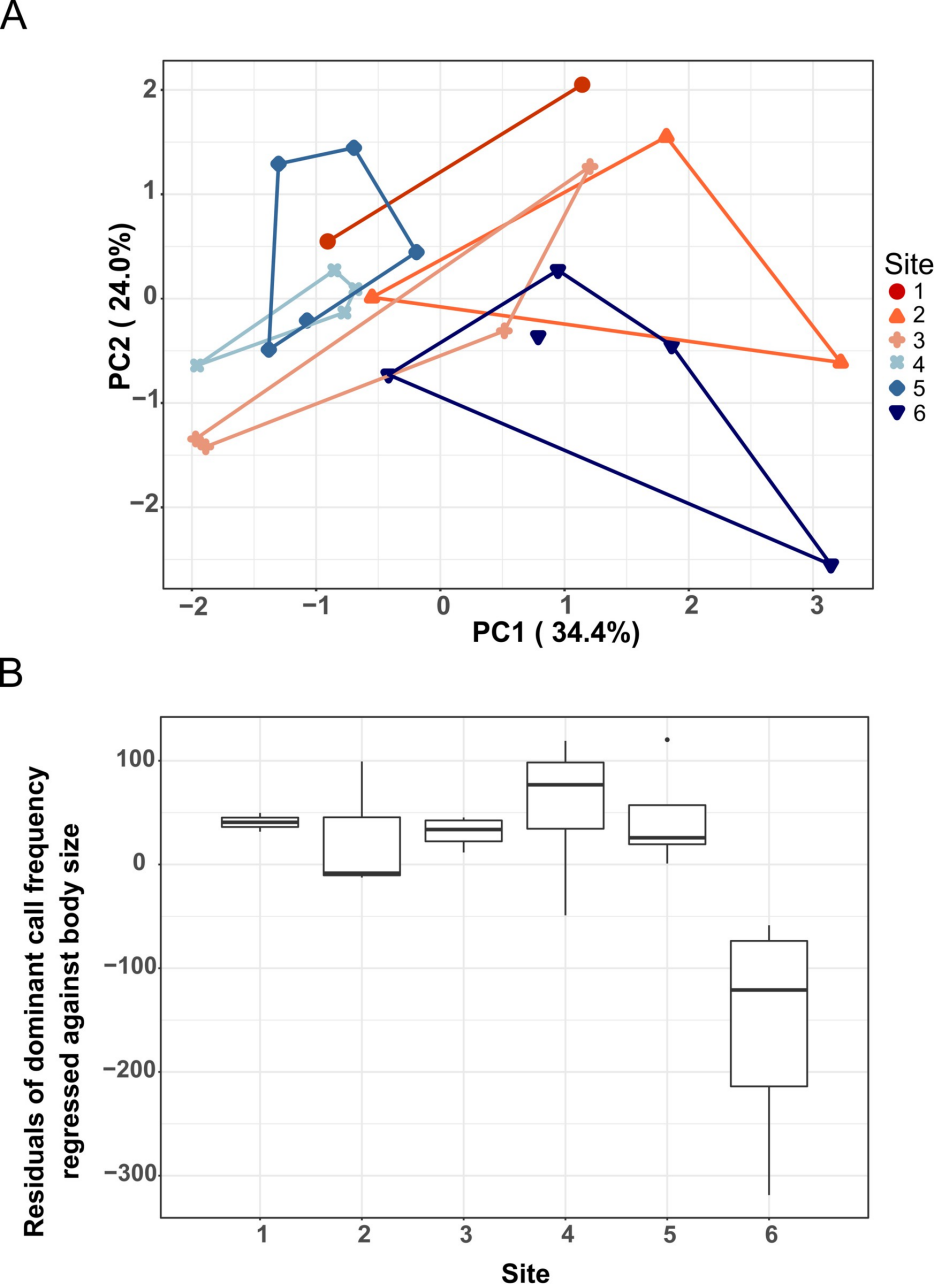
Analyses of call parameters of *Mantidactylus bellyi*. A] Principal Component Analysis (PCA) of the temporal and spectral call parameters, corrected for body temperature (T_b_) for temporal call characters and body size (SVL) for dominant call frequency by regression. Convex hulls were drawn for call scores of specimens belonging to different sites on the first two principal components. Each dot symbolizes average values of multiple calls per individual. The colors correspond to those in Fig 2A. B] Variation of the residuals of dominant call frequency regressed against body size of calling *Mantidactylus bellyi* between different study sites in Montagne d’Ambre National Park.

After removing size and body temperature effects from the original data, PCA visualization revealed a weak differentiation between calls from different sites. Specifically, sites 4 and 5 (with largely overlapping values) had lower values in Principal Component 1 (PC1) than most calls from the other sites, and most calls from site 6 had relatively low values of PC2 (Fig 4A). Yet, no significant differences were seen between sites in either PC1 or PC2 values (ANOVA, P > 0.05 for all tests). The residuals of the analyzed temporal call parameters did not show significant differences among the six sites (ANOVA, P > 0.05 for all tests), confirming the specimens as conspecific animals, and justifying comparisons of population-level differentiation within one species. Similarly, differences in temporal call parameters between the genetic clusters were not significant for K = 2–3 (ANOVA, P > 0.05 for all tests).

The residuals of dominant call frequency were different between the different sampled sites (ANOVA, F = 6.49, P < 0.01), among which individuals located in the western slope of the massif (site 6) displayed significantly lower dominant frequency in comparison to other sites (Fig 4B). Dominant call frequencies recorded at site 6 were even more deviating from those of the sites 4 and 5 (Tukey’s HSD, P < 0.01) despite their close geographic proximity, in comparison with those of the relatively distant sites 1 and 2 (Tukey’s HSD, P < 0.05). Dominant call frequency at site 6 also significantly diverged from that at site 3 (Tukey’s HSD, P < 0.01). However, the residuals of dominant call frequency were significantly different between the two genetic clusters for the preferred K = 2 model (ANOVA, F = 6.12, P = 0.02), but similar between the three genetic clusters for the preferred K = 3 models (ANOVA, F = 2.96, P = 0.08).

### Morphological variation

Body size of *M*. *bellyi* was between 27–42 mm in males and 25–54 mm in females (S4 Table in S1 File). Except for tympanum diameter (Spearman’s rank correlation test, rho = 0.06, df = 327, S = 5592242, P > 0.05), all other morphological measurements were positively correlated with SVL (Spearman’s rank correlation test, rho < 0.71, df = 327, P < 0.001).

**Fig 5:**
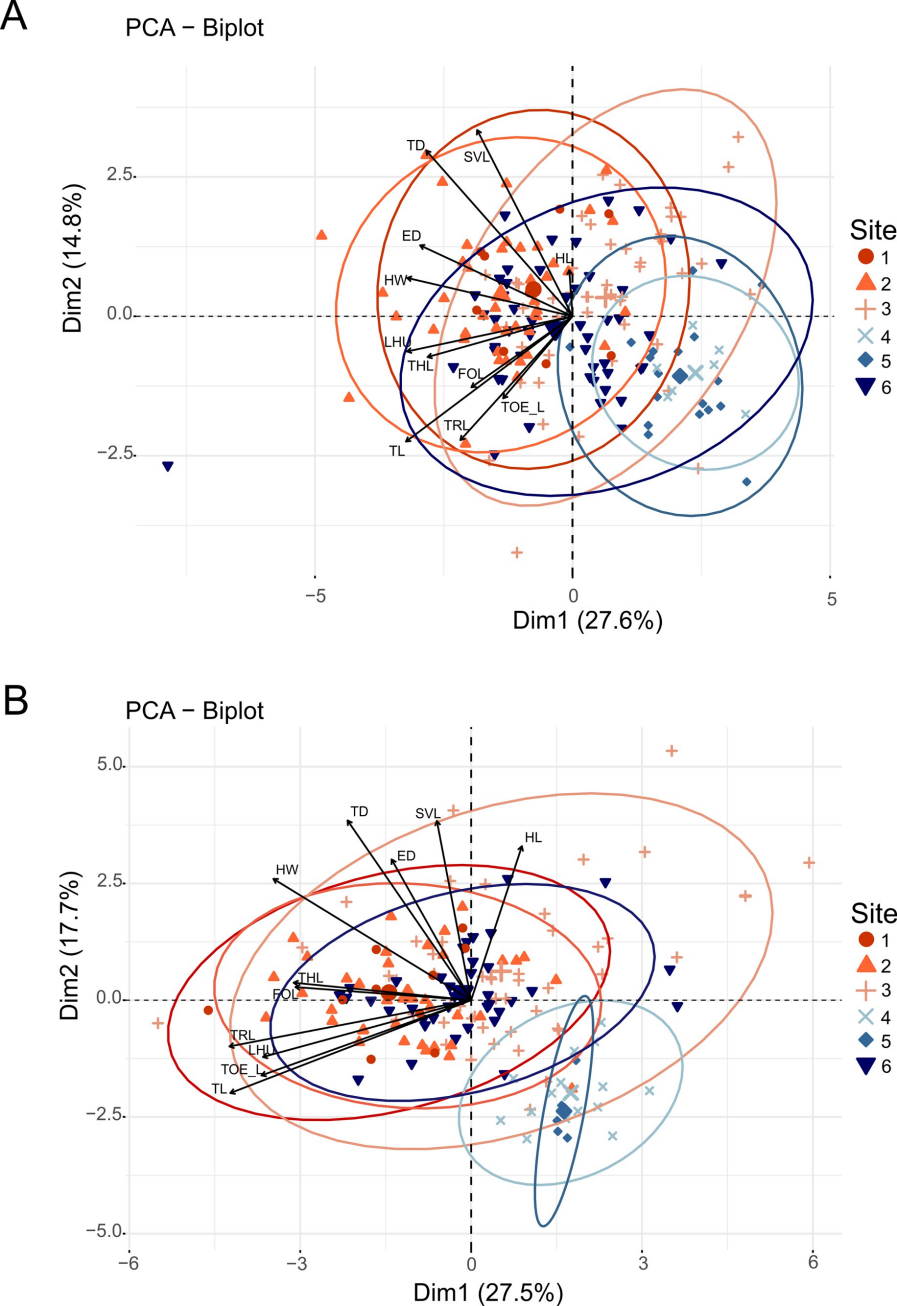
Multivariate analysis of the morphology of *Mantidactylus bellyi*. A] and B] show the variation of the residuals of morphological variables A] in males and B] in females between the six sites, corrected for the effect of size by regression, except SVL. Each small point symbolizes an individual’s sampling, with bigger dots representing the group mean point. Ellipses correspond to the 95% confidence interval. The colors refer to those in Fig 2A. We used the residual values of the variables for the analysis, except for SVL. The figure shows a detachment of individuals from sites 4 and 5 from the rest of the individuals in either case. SVL = snout–vent length, HL = head length, HW = maximum head width, ED = horizontal eye diameter, TD = tympanum diameter, LHU = humerus length, FOL = forearm length, THL = thigh length, TL = tibia length, TRL = tarsus length, TOE_L = length of the third toe.

After removing the size effect, we tested for variation of all morphological variables and the non-transformed SVL of *M*. *bellyi* between the six geographically distant sites. Overall, significant differences were observed in almost all morphological variables of *M*. *bellyi* between the different sampled sites as reported in Fig 5A and 5B, except for the length of the third toe (TOE_L) of males and the thigh length (THL) of females (Table 1). Pairwise comparisons between sites revealed close resemblance between morphology of individuals from sites 1 and 2 (S5 Table in S1 File), while individuals in site 3 more closely resembled individuals from site 1 than from site 2 (S5 Table in S1 File, Fig 5A and 5B). A total resemblance of the morphological measurements of individuals from site 4 (Lac Maudit) and site 5 (Grand Lac) was also observed. These observations were also illustrated in Fig 5A and 5B, where individuals in site 4 and site 5 formed a more similar group in terms of morphology, but were differentiated from the other sites by their relatively small body measurements (S5 Table in S1 File). To some degree, subpopulations living on the western slope (site 6) resembled individuals from sites 1 and 2, as they only differed by snout-vent-length, tympanum diameter and humerus length (S5 Table in S1 File).

**Table 1:** Comparisons of morphological variables between the six sites and between the genetic subpopulation clusters (K = 2 and K = 3 STRUCTURE models) based on ANOVA.

		6 sites	K = 3	K = 2
**SVL**	All	F = 25.96, ****	F = 36.72, ****	F = 4.48, *
	M	F = 31.53, ****	F = 42.42, ****	F = 4.28, *
	F	F = 24.16, ****	F = 15.16, ****	F = 4.27, *
**HL**	All	F = 15.13, ****	F = 8.04, ***	F = 9.77, **
	M	F = 5.40, ***	F = 2.98, P = 0.05	F = 1.88, P = 0.17
	F	F = 10.1, ****	F = 5.46, **	F = 8.54, **
**HW**	All	F = 9.87, ****	F = 9.75, ****	F = 7.26, **
	M	F = 4.07, **	F = 3.56, *	F = 0.28, P = 0.60
	F	F = 7.49, ****	F = 12.01, ****	F = 11.69, ***
**ED**	All	F = 8.58, ****	F = 9.94, ****	F = 4.39, *
	M	F = 6.15, ****	F = 8.97, ***	F = 1.56, P = 0.21
	F	F = 4.36, **	F = 2.85, P = 0.06	F = 2.94, P = 0.09
**TD**	All	F = 18.62, ****	F = 6.94, **	F = 0.90, P = 0.34
	M	F = 20.46, ****	F = 17.89, ****	F = 0.15, P = 0.70
	F	F = 21.96, ****	F = 10.23, ****	F = 0.95, P = 0.33
**LHU**	All	F = 37.4, ****	F = 2.13, P = 0.12	F = 1.96, P = 0.16
	M	F = 20.79, ****	F = 2.02, P = 0.14	F = 0.08, P = 0.77
	F	F = 17.51, ****	F = 2.13, P = 0.12	F = 4.27, *
**FOL**	All	F = 5.85, ****	F = 1.91, P = 0.15	F = 0.68, P = 0.41
	M	F = 2.60, *	F = 0.90, P = 0.41	F = 0.03, P = 0.88
	F	F = 4.19, **	F = 1.35, P = 0.26	F = 1.25, P = 0.27
**THL**	All	F = 6.87, ****	F = 9.32, ***	F = 0.55, P = 0.46
	M	F = 8.29, ****	F = 11.14, ****	F = 0.40, P = 0.53
	F	F = 1.58, p = 0.17	F = 2.26, P = 0.11	F = 1.89, P = 0.17
**TL**	All	F = 9.41, ****	F = 3.16, *	F = 1.59, P = 0.21
	M	F = 6.05, ****	F = 3.38, *	F = 3.83, P = 0.05
	F	F = 4.11, **	F = 0.63, P = 0.53	F = 0.00, P = 0.98
**TRL**	All	F = 5.89, ****	F = 2.22, P = 0.11	F = 0.80, P = 0.37
	M	F = 3.60, **	F = 2.11, P = 0.13	F = 0.22, P = 0.64
	F	F = 2.79, *	F = 1.40, P = 0.24	F = 2.84, P = 0.09
**TOE L**	All	F = 3.58, **	F = 0.57, P = 0.56	F = 0.66, P = 0.42
	M	F = 1.78, P = 0.12	F = 0.20, P = 0.82	F = 0.39, P = 0.53
	F	F = 2.33, *	F = 0.58, P = 0.56	F = 0.23, P = 0.63

Original values were regressed against SVL before comparisons, except SVL. P-values and F are given in the results. Significant differences were marked with * for P < 0.05,

** for P < 0.01,

*** for P < 0.001, and

**** for P < 0.0001.

SVL = snout–vent length, HL = head length, HW = maximum head width, ED = horizontal eye diameter, TD = tympanum diameter, LHU = humerus length, FOL = forearm length, THL = thigh length, TL = tibia length, TRL = tarsus length, TOE_L = length of the third toe. All = measurements of males and females, M = measurements of males only, F = measurements of females only.

When comparing the genetic subpopulation clusters for K = 2–3 STRUCTURE models, morphological divergences were more obvious when comparing three genetic subpopulations than when comparing two genetic subpopulations (Table 1). In all cases, SVL differed between the genetic subpopulation clusters. We detected differentiation in residual values of head length and head width (for K = 2 and K = 3) and eye diameter, tympanum diameter, thigh length and tibia length (for K = 3).

## Discussion

### Genetic diversity and population structure of *Mantidactylus bellyi* in MANP

Even though tropical regions comprise Earth’s greatest biodiversity hotspots [14, 93–95], investigations on ecological speciation are biased towards temperate regions and poorly explored in the tropics [96]. In this study, we tested for gene flow between individuals of the tropical frog *Mantidactylus bellyi* in the Montagne d’Ambre National Park (MANP), a site where the species is broadly distributed across heterogeneous environmental conditions. We know that montane habitats promote genetic isolation through elevational gradients and/or isolation by distance among vertebrates [4, 97–99]. Although we were able to confirm low mitochondrial differentiation within this species in our study area, microsatellite data yielded an optimal cluster solution of three subpopulations on the massif in three of our four STRUCTURE models (Fig 1 and S2 Table in S3 File). Admixture + LOCPRIOR models even detected signals of a possibly more fine-scale subdivision into up to five genetic clusters, which agrees with an overall hypothesis of limited gene flow among sites in this species. Given the spatial distribution of the three genetic clusters preferred by most models, the propensity for gene flow in *M*. *bellyi* appears to be related to geographical proximity, though not entirely; the geographic distance between sites 1 and 3 (both genetic cluster 1) is similar to that between site 3 and 4 (clusters 1 and 2), and much more than between sites 4 and 5 (genetic clusters 2 and 3). This implies a more complex relationship than pure isolation-by-distance. Here, the eclectic environmental conditions in Montagne d’Ambre also appear to be acting on the population structure of *M*. *bellyi*. Because the tadpoles of *M*. *bellyi* are susceptible of being carried downstream after hatching [100], we suspect the mono-directional down-slope flow of the streams in Montagne d’Ambre could be an important factor in gene flow between these semi-aquatic animals. This could explain the grouping pattern of the genetic cluster 2 (site 4 + site 6) since site 4 (Lac Maudit) drains largely to the West slope. The other sites drain to the East (site 5) and North-East (sites 1–3). The genetic clustering of these populations seen in some models (Fig 2) may suggest greater connectivity between these drainage systems than either has with the western drainage.

### Environmental ranges as physical barriers for adaptive genetic divergence

Species can often readily adapt to different environments [101–103] and elevations [104, 105]. Our study site, Montagne d’Ambre National Park, is largely covered by medium-elevation moist evergreen forest, but also includes heterogeneous forest types from moist semi-deciduous forest at low elevation to montane ericoid thicket at the summit [17]. The western slope is formed by dry deciduous forest, and areas have been disturbed by anthropogenic activities so that modified vegetation such as moist secondary thickets and secondary prairies can be found [17]. When barriers to gene flow are ecology-based, environmental conditions become crucial to the formation of new species [13, 103, 106, 107]. Our investigation of the influence of environment on genetic diversity of *Mantidactylus bellyi* on the massif, based on 13 environmental variables of its breeding streams and the forest areas surrounding them, yielded significant differences between the habitat characteristics of the genetic clusters 1 and 2, and 1 and 3. These differences were primarily in the mean depth and width of waters. The sites 4 and 6 (western site) also had the shallowest and the narrowest streams, coinciding with the molecular clustering of *M*. *bellyi* inhabiting them in some of our models. This reveals that there are indeed environmental differences between the areas inhabited by genetic clusters in *M*. *bellyi* in MANP in addition to the geographic distance between them and suggests the possibility for ecological specialization among subpopulations of these semi-aquatic frogs in physical microhabitat. However, the four physico-chemical water variables sampled here are insufficient to assess if genetic isolation may also be linked to these environmental properties. Other variables such as luminosity, flow rate, permanence and water temperature could certainly also affect habitat preference of this species and should be integrated in future studies. Temperature may be of particular importance, as body temperature of this species strongly parallels thermal changes of its immediate surroundings [61].

Individuals from near the summit (site 5) experience the most disparate ecological conditions. The vegetation structural profile of site 5 presented a tree layer noticeably contrasting with other sites by the existence of the shortest emerging trees, lowest total vegetation cover and short canopy height. Indeed, this area has a distinctive vegetation type and humid tropical microclimate [24, 108]. Frogs from the summit are also subjected to the highest body temperature variance [61]. Altogether, these characteristics could reinforce isolation of *M*. *bellyi* living at the summit. However, this population was grouped with the low-elevation cluster in one of our STRUCTURE analyses (Fig 2E). Why it would be less differentiated than animals that lie between it and the low-elevation cluster is unclear, and we suspect that the K = 3 (or K = 4) clustering, in which this relation is not apparent, reflects more closely the true relations among subpopulations. This would also imply a significant role of drainage system and connectivity in setting up isolation among populations.

### Bioacoustic divergence

Acoustic signals for mating are useful for anuran species delimitation and recognition as they could drive population differentiation and speciation [109–111]. Even though calls are mainly species-specific, intraspecific subtle differences in quantitative call variables or in call structure occur [112, 113]. Within the same population, body size, individual recognition and physical and physiological handicaps are reportedly the major determinants of intraspecific variation [87]. Consistent with other anurans [86], dominant frequency of the *M*. *bellyi* population of MANP was under morphological constraint, i.e. inversely correlated with body size. After removing temperature and size effects, we failed to find strong differences in temporal call properties among genetic clusters. This supports the assumption of a species-specific call characterizing frogs from all sites studied.

Dominant call frequency sometimes exhibits population variation, as has previously found in other anurans such as Darwin’s frog, *Rhinoderma darwinii* [114]. Such patterns between the call characters and the genetic diversity of *M*. *bellyi* were only found when comparing the dominant call frequencies under the two subpopulations (K = 2) model (site 5 lumped with sites 1–3). This is most probably due to the lower dominant call frequency range in calls from individuals inhabiting the western slope of the massif (site 6) compared to other individuals on MANP. While we might expect highest concordance between individuals of site 4 and 6 in their spectral acoustic signal based on their genetic structure and geographic proximity, they presented the most divergent dominant call frequencies. Because of relatively small sample sizes (N = 2–5) and methodological difficulties of objectively assessing dominant frequency in frogs with pulsed calls, we cannot ascertain the biological relevance of the encountered pattern, which might be related to environmental differences experienced by the frogs on the western slope (with the highest total vegetation cover value of 41%) given that lower call frequencies are less attenuated in any environment and travel longer distances than higher frequencies [115].

As previously stated, an important factor to consider is the possibility of unidirectional gene flow. Site 6 lies not only to the west of site 4 (Lac Maudit), but also below it. Run-off from the lake and surrounding areas flows west-wards down a number of streams, including those we surveyed. *Mantidactylus bellyi* adults, and still more likely their tadpoles, may be swept annually down-slope by the rains, which can be torrential. Re-ascending the slope would be a much greater challenge. Thus, gene flow is likely to go largely in a single direction, i.e. down-stream, in this system. Such down-stream dispersal would be expected to homogenize the down-stream populations with the up-stream ones across the neutral regions of the genome. For regions under strong local selection, dilution of the gene pool would weaken, but not necessarily prevent, adaptation in the down-stream population, such as ecological adaptation or sexual selection. This might, therefore, be one factor explaining the bioacoustic differentiation we observe in the absence of genetic differentiation in *M*. *bellyi* from the west slope of MANP.

### Morphological divergence

Factors driving morphological similarity between species include diet [116], behavior [117], physiological function [118, 119], and environmental characteristic [120]. We found that intraspecific genetic diversity was not consistent with morphological divergence in *M*. *bellyi*. After removing the size effect, significant divergence was observed in almost all morphological variables between the different sites (Table 1). Differences in SVL were coincident with molecular identity of the genetic subpopulation clusters, and in general morphological divergence was evident among the three populations recovered in most STRUCTURE analyses (Table 1). However, genetic clustering was not always consistent with morphological similarity. For example, individuals from sites 1 and 2 were practically indistinguishable, but only moderately similar to those from site 3 (S5 Table in S1 File), despite these sites clustering together in most of our genetic analyses. In contrast, the genetically differentiated summit population (site 5) closely resembled the population from site 4 in morphology. In this latter case, we suspect that elevational pressures may have shaped body size; these sites are the highest sampled by us, and they differ from all other subpopulations by their smaller body size (snout–vent length). Indeed, body size of *M*. *bellyi* positively correlates with its body temperature and we know body temperature in elevated sites of Montagne d’Ambre is colder and shows the highest variance [61].

## Conclusions

Our analysis discovered a clear population-level differentiation within the montane frog *Mantidactylus (Brygoomantis) bellyi* of MANP. Bioacoustic differences, albeit relatively weak, were observed as well; they were not fully consistent with genetic clusters but partly related to body size differences. Morphology, and in mantellid frogs especially body size, plays an important role in determining dispersal ability and physiological tolerance, and thus gene flow [37, 60]. Overall, *M*. *bellyi* is not a particularly small-sized mantellid, and the low amount of haplotype divergence in the *16S* rRNA gene among populations from Montagne d’Ambre and other localities such as Ankarana, revealed by our study, suggests this species is able to maintain gene flow over rather large distances. This makes the distinct subpopulation structure at Montagne d’Ambre even more intriguing. The possible combined role of isolation-by-distance and hypothesized isolation-by-ecology (microhabitat) might offer a partial explanation of the encountered pattern, but it remains difficult to understand the causes underlying the almost total lack of gene flow among neighboring sites such as 4/6 versus 5. To solve this conundrum, future studies should (i) map in some detail the presence of suitable water bodies across the mountain since these are sometimes very small and the available data do not allow to ascertain their presence from satellite imagery, and areas with a low density of streams may offer a higher resistance to dispersal of *M*. *bellyi* individuals; (ii) assess variables directly related to microhabitat and breeding sites to test the hypothesis of genetic isolation triggered by habitat-based divergent selection and (iii) include a wider geographical sampling to understand if genetic clusters are locally restricted, microendemic subpopulations that may have diverged in situ and adapted to local environmental conditions such as elevation and temperature, or are more widespread and coincidentally form a contact zone near the summit of Montagne d’Ambre.

## Supporting information

S1 File Supporting file for morphological results, gathering the S4 and S5 Tables. S4 Table gives the summary table of the minimum, the maximum and the mean values ± standard deviation of *Mantidactylus bellyi*’s morphological measurements and the number of individuals surveyed for the six different sites, represented as Min–Max (Mean ± SD, N). S5 Table gives the Tukey’s Honest Significant Difference test for pairwise comparison among sites of the morphological variables. Original values were regressed against SVL before comparisons, except SVL. Significant differences were marked with * when p < 0.05, ** when p < 0.01, and *** when p < 0.001. SVL = snout–vent length, HL = head length, HW = maximum head width, ED = horizontal eye diameter, TD = tympanum diameter, LHU = humerus length, FOL = forearm length, THL = thigh length, TL = tibia length, TRL = tarsus length, TOE_L = length of the third toe. All = measurements of males and females, M = measurements of males only, F = measurements of females only.
(PDF)

S2 File Supporting file for acoustic analyses and results, gathering the S1 Fig and the S3 Table. S1 Fig gives the graphic representations of the advertisement call of *Mantidactylus bellyi* (SRTIS 113) showing the different call parameters. A] Spectrogram, B] Oscillogram, C] Zoomed view of the oscillogram D] Call spectrum for visualizing the dominant frequency. S3 Table gives the summary table of the minimum, the maximum, the mean values ± standard deviation of the males of *M*. *bellyi* call parameters, and the number of analyzed calls for the six different sites, represented as Min–Max (Mean ± SD, N).
(PDF)

S3 File Supporting file for genetic analyses and results, gathering the S1 and S2 Tables, and the S2 Fig (N = 236 individuals). S1 Table gives the list of forward (Fwd) and reverse (Rev) primers for seven newly established microsatellite markers used for *Mantidactylus bellyi*. Repeat count are from the initial library. Percentage of missing data, numbers and length ranges of alleles refer to the entire dataset. Length range (inferred bp) includes primers and linker. S2 Table gives the inference of the population structure of *M*. *bellyi* based on Bayesian analysis for K = 1 to K = 7 for four models. Ad = Admixture model, AdLOCPRIOR = Admixture model with LOCPRIOR information, NoAd = No admixture model, NoAdLOCPRIOR = No admixture model with LOCPRIOR information. K = number of assumed subpopulations; Reps = number of MCMC iterations; ΔK = ad hoc statistic based on the change in the log probability data between successive K values. The most likely K is highlighted in bold. S2 Fig gives the genetic cluster assignment of the sampled populations of *M*. *bellyi* in Montagne d’Ambre. A–E] Individual assignments of *M*. *bellyi* to genetic clusters as inferred by STRUCTURE from a data set of seven microsatellites. A–C] The clustering scenarios of assumed subpopulations K = 4–6 were depicted for the models showing highest ΔK, i.e. A] Admixture model with LOCPRIOR information for K = 4, B] Admixture model for K = 5, and C] Admixture model for K = 6. D–E] The clustering scenarios were depicted for no admixture models with LOCPRIOR information of assumed subpopulations D] K = 5, and E] K = 6.
(PDF)
