# Supplementary material for: Population diversification in the frog Mantidactylus bellyi on an isolated massif in northern Madagascar based on genetic, morphological, bioacoustic and ecological evidence
Source: PLoS One. 2022 Mar 31;17(3):e0263764. doi: 10.1371/journal.pone.0263764 (PMC8970393; doi:10.1371/journal.pone.0263764)
Supplement: S1 File — S4 Table gives the summary table of the minimum, the maximum and the mean values ± standard deviation of Mantidactylus bellyi’s morphological measurements and the number of individuals surveyed for the six different sites, represented as Min–Max (Mean ± SD, N). S5 Table gives the Tukey’s Honest Significant Difference test for pairwise comparison among sites of the morphological variables. Original values were regressed against SVL before comparisons, except SVL. Significant differences were marked with * when p < 0.05, ** when p < 0.01, and *** when p < 0.001. SVL = snout–vent length, HL = head length, HW = maximum head width, ED = horizontal eye diameter, TD = tympanum diameter, LHU = humerus length, FOL = forearm length, THL = thigh length, TL = tibia length, TRL = tarsus length, TOE_L = length of the third toe. All = measurements of males and females, M = measurements of males only, F = measurements of females only. (PDF) [file pone.0263764.s001.pdf]

S4 Table

Sites		1	2	3	4	5	6	MANP
**SVL (mm)**	All	35.7–49.7 (40.4 ± 4.3, 19)	31.5–53.9 (40.7 ± 4.8, 80)	24.7–47.8 (39.0 ± 5.1, 97)	28.4–36.3 (31.7 ± 2.1, 22)	25.5–40.5 (30.6 ± 3.2, 23)	27.2–50.3 (38.9 ± 5.2, 88)	24.7–53.9 (38.4 ± 5.6, 329)
	M	36.1–42.1 (38.0 ± 2.4, 9)	31.5–41.7 (37.9 ± 1.9, 44)	27.7–40.9 (35.5 ± 2.6,48)	28.4–33.4 (30.5 ± 1.8, 7)	27.8–33.3 (30.6 ± 1.6, 18)	27.2–41.3 (35.3 ± 2.9, 50)	27.2–42.1 (35.5 ± 3.3, 176)
	F	35.7–49.7 (42.6 ± 4.6, 10)	35.7–53.9 (44.1 ± 5.1, 36)	24.7–47.8 (42.4 ± 4.6, 49)	29.3–36.3 (32.3 ± 2.0, 15)	25.5–40.5 (30.7 ± 6.6, 5)	32.9–50.3 (43.6 ± 3.7, 38)	24.7–53.9 (41.7 ± 5.8, 153)
**HL (mm)**	All	13.1–17.0 (14.8 ± 1.2, 19)	11.9–18.1 (14.4 ± 1.5, 80)	9.9–22.6 (15.1 ± 2.3, 97)	10.1–12.8 (11.3 ± 0.8, 22)	9.2–14.2 (11.3 ± 1.2, 23)	11.2–19.3 (14.1 ± 1.7, 88)	9.2–22.6 (14.1 ± 2.1, 329)
	M	13.1–15.8 (14.1 ± 1.0, 9)	11.9–16.4 (13.7 ± 1.1, 44)	9.9–18.5 (13.9 ± 1.6,48)	10.1–12.0 (10.9 ± 0.7, 7)	10.3–13.8 (11.4 ± 0.9, 18)	11.2–16.0 (13.2 ± 1.0, 50)	9.9–18.5 (13.3 ± 1.5, 176)
	F	14.0–17.0 (15.4 ± 1.1, 10)	12.0–18.1 (15.2 ± 1.5, 36)	10.9–22.6 (16.4 ± 2.3, 49)	10.4–12.8 (11.6 ± 0.8, 15)	9.2–14.2 (11.1 ± 2.0, 5)	12.1–19.3 (15.4 ± 1.7, 38)	9.2–22.6 (15.2 ± 2.3, 153)
**HW (mm)**	All	15.0–19.3 (16.9 ± 1.2, 19)	14.7–21.5 (17.1 ± 1.6, 80)	11.3–20.4 (16.4 ± 1.9, 97)	10.1–14.8 (12.9 ± 1.0, 22)	10.4–15.8 (12.9 ± 1.3, 23)	12.7–19.5 (16.3 ± 1.7, 88)	10.1–21.5 (16.1 ± 2.1, 329)
	M	15.0–17.6 (16.2 ± 0.8, 9)	14.7–18.2 (16.3 ± 1.0, 44)	11.3–17.5 (15.2 ± 1.1, 48)	10.1–13.6 (12.6 ± 1.3, 7)	11.6–14.6 (13.1 ± 1.0, 18)	12.7–17.4 (15.2 ± 1.1, 50)	10.1–18.2 (15.2 ± 1.5, 176)
	F	15.4–19.3 (17.5 ± 1.2, 10)	15.7–21.5 11.4–20.4 (18.1 ± 1.7, 36)	11.4–20.4 (17.6 ± 1.9, 49)	11.6–14.8 (13.0 ± 0.9, 15)	10.4–15.8 (12.4 ± 2.3, 5)	13–19.5 (17.7 ± 1.3, 38)	10.4–21.5 (17.1 ± 2.3, 153)
**ED (mm)**	All	4.6–6.9 (5.9 ± 0.7, 19)	4.8–8.5 (5.8 ± 0.6, 80)	3.7–7.1 (5.5 ± 0.7, 97)	3.7–5.3 (4.2 ± 0.4, 22)	3.5–5.3 (4.2 ± 0.5, 23)	4.2–7.2 (5.5 ± 0.6, 88)	3.5–8.5 (5.4 ± 0.8, 329)
	M	4.7–6.1 (5.6 ± 0.5, 9)	4.8–8.5 (5.6 ± 0.7, 44)	4.2–5.9 (5.1 ± 0.4, 48)	3.8–4.4 (4.1 ± 0.3, 7)	3.5–5.3 (4.2 ± 0.4, 18)	4.2–6.5 (5.1 ± 0.5, 50)	3.5–8.5 (5.1 ± 0.7, 176)
	F	4.6–6.9 (6.1 ± 0.7, 10)	5.0–7.3 (5.9 ± 0.6, 36)	3.7–7.1 (5.8 ± 0.7, 49)	3.7–5.3 (4.2 ± 0.4, 15)	3.6–5.1 (4.2 ± 0.7, 5)	4.7–7.2 (5.9 ± 0.5, 38)	3.6–7.3 (5.7 ± 0.8, 153)
**TD (mm)**	All	3.0–5.7 (4.4 ± 0.8, 19)	3.3–6.5 (4.8 ± 0.8, 80)	2.0–5.8 (4.0 ± 0.8, 97)	2.3–4.7 (3.2 ± 0.7, 22)	2.1–4.7 (3.6 ± 0.7, 23)	2.9–6.1 (4.4 ± 0.7, 88)	2.0–6.5 (4.3 ± 0.9, 329)
	M	4.0–5.7 (5.1 ± 0.6, 9)	4.1–6.5 (5.4 ± 0.5, 44)	2.4–5.8 (4.5 ± 0.7, 48)	3.6–4.7 (4.0 ± 0.4, 7)	2.7–4.7 (3.9 ± 0.4, 18)	3.4–6.1 (4.8 ± 0.6, 50)	2.4–6.5 (4.8 ± 0.8, 176)
	F	3.0–4.8 (3.9 ± 0.6, 10)	3.3–5.0 (4.1 ± 0.5, 36)	2.0–4.5 (3.6 ± 0.5, 49)	2.3–3.8 (2.8 ± 0.4, 15)	2.1–3.1 (2.5 ± 0.4, 5)	2.9–4.6 (3.9 ± 0.4, 38)	2.0–5.0 (3.7 ± 0.6, 153)
**LHU (mm)**	All	9.7–14.4 (11.8 ± 1.2, 19)	9.0–15.7 (11.8 ± 1.4, 80)	6.5–12.7 (9.4 ± 1.5, 97)	5.3–9.4 (7.6 ± 0.9, 22)	5.7–11.0 (7.9 ± 1.1, 23)	7.4–14.0 (10.3 ± 1.5, 88)	5.3–15.7 (10.2 ± 1.9, 329)
	M	9.7–12.5 (11.2 ± 0.9, 9)	9.0–12.9 (11.1 ± 0.9, 44)	6.5–11.5 (8.7 ± 1.2, 48)	6.1–9.4 (7.7 ± 1.0, 7)	7.1–8.9 (7.8 ± 0.6, 18)	7.5–12.5 (9.7 ± 1.2, 50)	6.1–12.9 (9.6 ± 1.6, 176)

	F	10.8–14.4 (12.3 ± 1.3, 10)	9.9–15.7 (12.7 ± 1.4, 36)	6.8–12.7 (10.2 ± 1.4, 49)	5.3–8.9 (7.6 ± 0.8, 15)	5.7–11.0 (8.1 ± 2.3, 5)	7.4–14.0 (11.2 ± 1.5, 38)	5.3–15.7 (10.8 ± 2.1, 153)
**FOL (mm)**	All	7.8–11.8 (9.4 ± 1.0, 19)	7.7–17.6 (9.4 ± 1.3, 80)	5.8–12.3 (8.5 ± 1.4, 97)	5.7–8.4 (6.8 ± 0.7, 22)	5.5–8.7 (6.8 ± 0.7, 23)	6.5–11.0 (8.7 ± 1.1, 88)	5.5–17.6 (8.6 ± 1.4, 329)
	M	7.8–9.2 (8.6 ± 0.5, 9)	7.7–17.6 (8.9 ± 1.5, 44)	5.8–9.8 (7.8 ± 1.0, 48)	6.0–7.1 (6.6 ± 0.4, 7)	6.1–7.6 (6.8 ± 0.4, 18)	6.5–9.6 (8.1 ± 0.7, 50)	5.8–17.6 (8.1 ± 1.2, 176)
	F	8.5–11.8 (10.1 ± 0.9, 10)	8.1–11.7 (10.0 ± 0.9, 36)	6.4–12.3 (9.2 ± 1.4, 49)	5.7–8.4 (6.9 ± 0.7, 15)	5.5–8.7 (6.7 ± 1.2, 5)	8.2–11 (9.6 ± 0.7, 38)	5.5–12.3 (9.2 ± 1.4, 153)
**THL (mm)**	All	17.0–23.4 (19.9 ± 1.9, 19)	16.0–26.0 (20.3 ± 2.1, 80)	13.9–25.5 (19.8 ± 2.3, 97)	13.0–17.8 (15.5 ± 1.3, 22)	12.9–18.5 (14.9 ± 1.3, 23)	11.3–24.3 (19.6 ± 2.5, 88)	11.3–26.0 (19.3 ± 2.7, 329)
	M	17.0–20.6 (18.5 ± 1.1, 9)	16.0–22.8 (19.1 ± 1.4, 44)	15.4–20.8 (18.4 ± 1.2, 48)	13.5–15.7 (14.9 ± 0.8, 7)	13.0–16.4 (14.9 ± 1.0, 18)	15.0–20.6 (18.2 ± 1.3, 50)	13.0–22.8 (18.0 ± 1.8, 176)
	F	17.5–23.4 (21.1 ± 1.7, 10)	18.4–26.0 (21.7 ± 1.9, 36)	13.9–25.5 (21.2 ± 2.4, 49)	13.0–17.8 (15.8 ± 1.4, 15)	12.9–18.5 (15.0 ± 2.3, 5)	11.3–24.3 (21.5 ± 2.5, 38)	11.3–26.0 (20.7 ± 2.9, 153)
**TL (mm)**	All	17.0–23.3 (19.8 ± 1.7, 19)	17.4–24.3 (20.0 ± 1.9, 80)	12.2–23.5 (18.2 ± 2.7, 97)	13.3–17.2 (15.1 ± 1.0, 22)	11.9–18.7 (14.6 ± 1.5, 23)	15.0–24.5 (19.0 ± 2.3, 88)	11.9–24.5 (18.5 ± 2.7, 329)
	M	17.0–19.6 (18.4 ± 0.8, 9)	17.4–20.6 (18.7 ± 0.8, 44)	12.2–20.2 (16.6 ± 2.0, 48)	13.3–15.3 (14.5 ± 0.8, 7)	11.9–16.2 (14.6 ± 1.1, 18)	15.0–19.5 (17.5 ± 1.1, 50)	11.9–20.6 (17.2 ± 1.8, 176)
	F	19.3–23.3 (21.0 ± 1.1, 10)	18.8–24.3 (21.6 ± 1.5, 36)	13.5–23.5 (19.7 ± 2.5, 49)	13.8–17.2 (15.4 ± 1.0, 15)	12.1–18.7 (14.7 ± 2.7, 5)	15.2–24.5 (21.1 ± 1.9, 38)	12.1–24.5 (20.0 ± 2.8, 153)
**TRL (mm)**	All	9.2–12.8 (10.9 ± 1.0, 19)	9.2–13.7 (10.9 ± 1.0, 80)	6.2–18.8 (9.9 ± 1.9, 97)	7.0–9.2 (8.0 ± 0.7, 22)	6.7–10.1 (7.9 ± 0.8, 23)	7.1–13.2 (10.1 ± 1.1, 88)	6.2–18.8 (10.0 ± 1.6, 329)
	M	9.2–11.2 (10.2 ± 0.6, 9)	9.2–11.2 (10.3 ± 0.5, 44)	6.2–17.3 (9.0 ± 1.6, 48)	7.0–8.8 (7.8 ± 0.6, 7)	6.7–8.9 (7.8 ± 0.7, 18)	7.1–10.5 (9.4 ± 0.7, 50)	6.2–17.3 (9.3 ± 1.30, 176)
	F	10.6–12.8 (11.6 ± 0.8, 10)	9.7–13.7 (11.6 ± 1.0, 36)	6.9–18.8 (10.7 ± 1.9, 49)	7.1–9.2 (8.1 ± 0.7, 15)	7.2–10.1 (8.2 ± 1.3, 5)	8.4–13.2 (11.0 ± 1.0, 38)	6.9–18.8 (10.7 ± 1.7, 153)
**TOE L (mm)**	All	14.9–23.7 (19.8 ± 2.0, 19)	10.3–24.2 (19.9 ± 2.2, 80)	7.9–28.8 (18.2 ± 2.8, 97)	13.4–17.4 (15.6 ± 1.0, 22)	13.1–18.4 (15.1 ± 1.3, 23)	1.6–29.8 (18.9 ± 3.0, 88)	1.6–29.8 (18.5 ± 2.9, 329)
	M	14.9–19.7 (18.3 ± 1.3, 9)	10.3–21.4 (18.7 ± 1.7, 44)	7.9–20.5 (16.7 ± 2.1, 48)	13.4–15.3 (14.7 ± 0.6, 7)	13.5–16.5(15.2 ± 1.0, 18)	1.6–20.0 (17.4 ± 2.7, 50)	1.6–21.4 (17.2 ± 2.3, 176)
	F	19.0–23.7 (21.3 ± 1.3, 10)	17.2–24.2 (21.3 ± 1.8, 36)	12.1–28.8 (19.7 ± 2.6, 49)	14.7–17.4 (16.0 ± 0.8, 15)	13.1–18.4 (14.8 ± 2.4, 5)	16.2–29.8 (20.9 ± 2.2, 38)	12.1–29.8 (20.0 ± 2.8, 153)

S5 Table

Sites		1-2	1-3	1-4	1-5	1-6	2-3	2-4	2-5	2-6	3-4	3-5	3-6	4-5	4-6	5-6
**SVL**	All	1.00	0.83	***	***	0.82	0.15	***	***	0.15	***	***	1.00	0.97	***	***
	M	1.00	0.05	***	***	*	***	***	***	***	***	***	1.00	1.00	***	***
	F	0.93	1.00	***	***	0.98	0.47	***	***	1.00	***	***	0.77	0.98	***	***
**HL**	All	0.57	0.09	0.23	0.73	0.97	***	0.86	1.00	0.71	***	***	***	0.95	0.30	0.92
	M	0.95	0.69	0.50	0.90	1.00	***	0.75	1.00	0.91	**	**	*	0.91	0.40	0.86
	F	0.67	0.22	0.70	0.90	0.95	***	1.00	1.00	0.93	***	0.05	***	1.00	0.95	0.99
**HW**	All	0.98	1.00	***	0.26	1.00	0.99	***	**	0.68	***	*	0.94	0.29	***	0.08
	M	1.00	1.00	0.09	0.63	1.00	0.83	**	*	0.97	*	0.31	1.00	0.62	*	0.16
	F	1.00	0.98	*	0.10	0.99	0.99	***	*	0.66	***	**	0.22	1.00	*	0.13
**ED**	All	0.90	0.26	***	***	0.27	0.45	***	***	0.49	**	*	1.00	0.98	**	*
	M	1.00	0.87	0.12	*	0.88	0.09	**	***	0.09	0.28	0.06	1.00	1.00	0.26	0.05
	F	0.15	0.29	***	0.08	0.24	0.99	0.06	0.77	1.00	*	0.58	1.00	1.00	*	0.67
**TD**	All	0.45	0.36	***	*	1.00	***	***	***	*	**	0.41	**	0.59	**	***
	M	0.72	0.14	*	***	0.89	***	***	***	***	0.43	**	0.13	0.98	*	***
	F	0.72	0.31	***	***	1.00	***	***	***	0.44	***	***	**	0.82	***	***
**LHU**	All	1.00	***	***	***	***	***	***	***	***	1.00	0.22	***	0.59	**	0.72
	M	1.00	***	*	**	0.10	***	**	***	***	0.97	0.74	***	1.00	0.43	0.13
	F	1.00	***	***	0.45	*	***	***	0.27	***	1.00	0.56	0.06	0.65	0.33	1.00
**FOL**	All	1.00	0.09	*	0.15	0.68	***	**	*	0.28	0.83	1.00	0.36	0.98	0.15	0.59
	M	0.92	0.97	0.89	0.97	1.00	*	0.24	0.15	0.56	0.99	1.00	0.70	1.00	0.74	0.82
	F	0.68	*	**	0.14	0.19	0.08	*	0.51	0.78	0.88	1.00	0.77	1.00	0.34	0.88
**THL**	All	0.95	0.49	0.58	0.30	0.88	0.68	*	**	1.00	***	***	0.87	1.00	*	**
	M	0.56	0.10	0.94	0.78	0.25	0.58	0.10	**	0.95	**	***	0.97	1.00	*	***
	F	1.00	1.00	0.74	0.83	1.00	0.98	0.45	0.73	1.00	0.16	0.51	0.94	1.00	0.54	0.79
**TL**	All	1.00	*	0.09	0.07	1.00	***	**	**	0.93	1.00	1.00	***	1.00	*	*
	M	0.99	0.50	0.73	0.59	1.00	***	0.19	*	1.00	1.00	1.00	**	1.00	0.26	*
	F	1.00	0.19	0.21	0.49	0.98	**	*	0.37	0.92	1.00	1.00	0.13	1.00	0.25	0.67
**TRL**	All	1.00	*	*	0.06	0.39	***	*	*	0.18	0.97	1.00	0.46	1.00	0.40	0.61
	M	1.00	0.52	0.71	0.38	0.99	*	0.38	*	0.76	1.00	0.99	0.30	1.00	0.80	0.27
	F	0.97	0.15	0.09	0.83	0.29	0.12	0.11	0.97	0.36	0.97	1.00	1.00	0.97	0.89	1.00
**TOE L**	All	1.00	0.16	0.66	0.64	0.98	**	0.51	0.48	0.97	0.99	0.99	0.05	1.00	0.82	0.80
	M	0.99	0.95	0.98	1.00	1.00	0.08	0.70	0.88	0.99	1.00	0.94	0.28	0.99	0.87	0.99
	F	0.95	0.14	0.52	0.43	0.83	0.14	0.82	0.69	1.00	0.99	1.00	0.35	0.99	0.95	0.82
