# Supplementary material for: Population diversification in the frog Mantidactylus bellyi on an isolated massif in northern Madagascar based on genetic, morphological, bioacoustic and ecological evidence
Source: PLoS One. 2022 Mar 31;17(3):e0263764. doi: 10.1371/journal.pone.0263764 (PMC8970393; doi:10.1371/journal.pone.0263764)
Supplement: S2 File — S1 Fig gives the graphic representations of the advertisement call of Mantidactylus bellyi (SRTIS 113) showing the different call parameters. A] Spectrogram, B] Oscillogram, C] Zoomed view of the oscillogram D] Call spectrum for visualizing the dominant frequency. S3 Table gives the summary table of the minimum, the maximum, the mean values ± standard deviation of the males of M. bellyi call parameters, and the number of analyzed calls for the six different sites, represented as Min–Max (Mean ± SD, N). (PDF) [file pone.0263764.s002.pdf]

S1 Fig

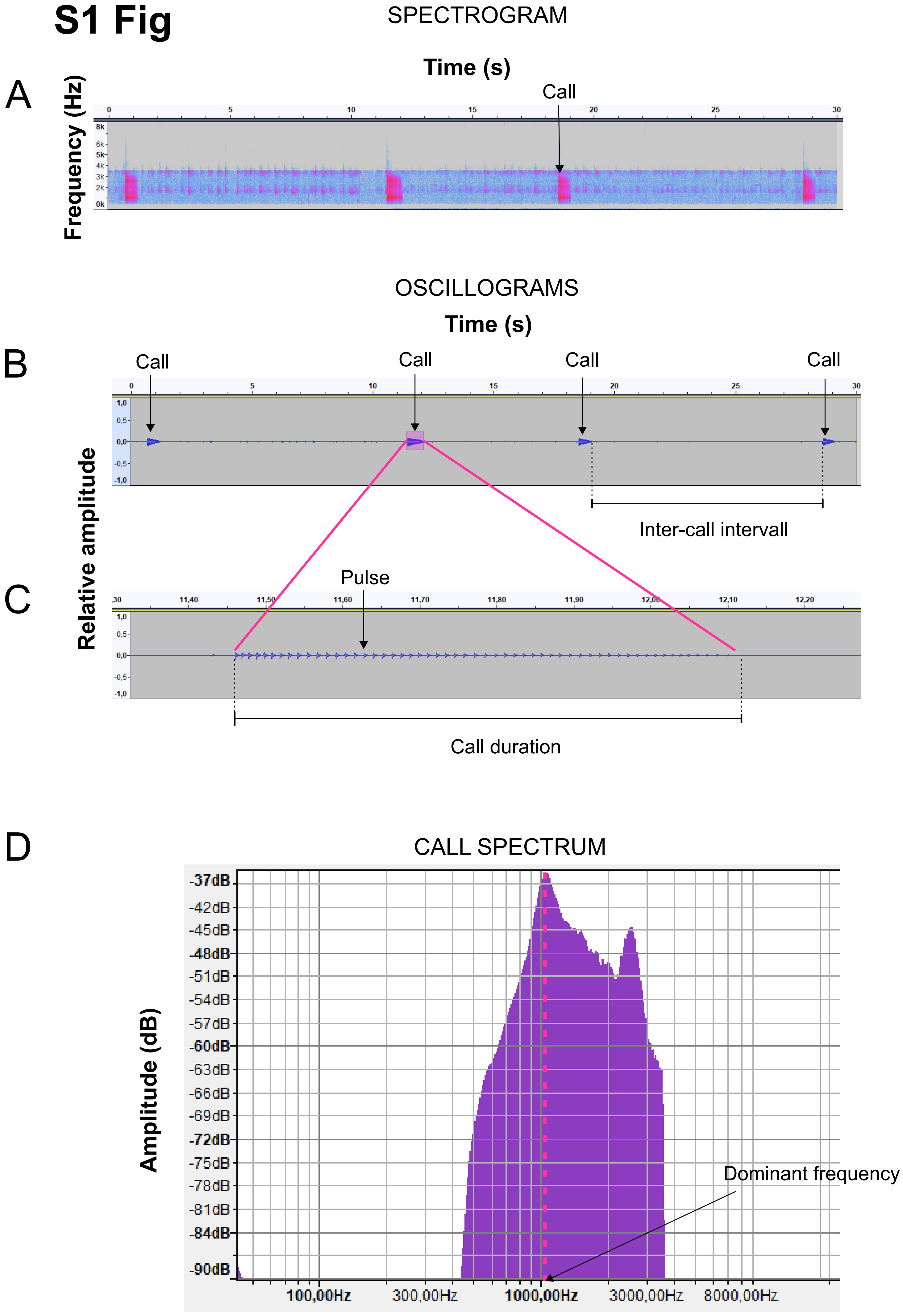
A
SPECTROGRAM
Time (s)
Frequency (Hz)
Call
B
OSCILLOGRAMS
Time (s)
Call
Call
Call
Call
Relative amplitude
Inter-call intervall
C
Pulse
Call duration
D
CALL SPECTRUM
Amplitude (dB)
Dominant frequency
-37dB
-42dB
-45dB
-48dB
-51dB
-54dB
-57dB
-60dB
-63dB
-66dB
-69dB
-72dB
-75dB
-78dB
-81dB
-84dB
-90dB
100,00Hz
300,00Hz
1000,00Hz
3000,00Hz
8000,00Hz

S3 Table

Sites	1	2	3	4	5	6	MANP
**Call duration (ms)**	442–697 (581.3 ± 102.8,7)	348–1595 (982.8 ± 433.5, 14)	307–1185 (627.9 ± 225.9, 34)	255–986 (521.1 ± 201.1, 66)	240–826 (505.3 ± 143.8, 72)	110–1599 (812.3 ± 356.7, 37)	110–1599 (608.7 ± 277.6, 230)
**Number of pulses**	60–83 (71.6 ± 8.8, 7)	30–157 (109.5 ± 38.0, 14)	20–122 (57.7 ± 29.1, 34)	21–90 (50.9 ± 20.5, 66)	23–92 (55.0 ± 16.8, 72)	13–218 (89.5 ± 40.0, 37)	13–218 (63.6 ± 31.3, 230)
**Duration of pulse (ms)**	7.0–8.9 (8.1 ± 0.6, 7)	7.0–11.6 (8.9 ± 1.5, 14)	5.8–16.4 (11.8 ± 2.3, 34)	7.7–12.7 (10.4 ± 0.8, 66)	6.8–12.7 (9.3 ± 1.5, 72)	5.6–11.8 (9.1 ± 1.5, 37)	5.6–16.4 (9.9 ± 1.8, 230)
**Inter-call interval (ms)**	23486–67151 (41591.8 ± 16388.9, 7)	27191–101870 (46654.6 ± 22520.3, 14)	440–87938 (27110.2 ± 23428.4, 34)	194–48504 (11768.6 ± 13394.2, 66)	358–56742 (18295.9 ± 13335.1, 72)	210–77549 (29213.4 ± 19305.6, 37)	194–101870 (21360.9 ± 19017.9, 230)
**Pulse rate (n/ s)**	112.1–142.5 (124.4 ± 10.1, 7)	86.2–143.1 (115.5 ± 19.7, 14)	31.1–171.2 (89.0 ± 22.4, 34)	78.7–130.3 (96.9 ± 7.6, 66)	78.8–146.7 (109.7 ± 17.1, 72)	85.1–178.9 (112.8 ± 20.5)	61.1–178.9 (104.3 ± 19.0, 230)
**Dominant frequency (Hz)**	1097–1210 (1145.4 ± 47.2, 7)	928–1181 (1107 ± 63.6, 14)	999–1437 (1098 ± 78.4, 34)	914–1473 (1225.6 ± 121.9, 66)	1070–1375 (1205.7 ± 62.8, 72)	857–1335 (977.4 ± 90.9, 37)	857–1473 (1150.9 ± 126.0, 230)
**Number of individuals**	2	3	4	4	5	5	23
