# Supplementary material for: Population diversification in the frog Mantidactylus bellyi on an isolated massif in northern Madagascar based on genetic, morphological, bioacoustic and ecological evidence
Source: PLoS One. 2022 Mar 31;17(3):e0263764. doi: 10.1371/journal.pone.0263764 (PMC8970393; doi:10.1371/journal.pone.0263764)

S1 Table

Marker	5'-3' primer sequence	Repeat	Repeat count	N alleles	Length range [bp]	Missing data [%]
**158602**	Fwd : GCAGAGAACTTGTTGGCCATAC Rev : GACCAGCTTCATATTTCTGGCC	ATCC	12	25	129-191	1.7
**27880**	Fwd : CACTATGCATGACTGGCACTAG Rev : ATCGTCTCCTGTGTCCTATGTG	AGAT	12	19	362-486	13.6
**16089**	Fwd : CAACGACTGCCAGATTTCCTAG Rev : TGCCCTAGTGAGACCTTTGAAC	ACAT	15	19	254-338	1.7
**31964**	Fwd : CTTTCTTGACCAACTCCACTCC Rev : CCCTCAGAAACTGCAGTGAATG	ACAG	14	31	370-498	18.2
**102671**	Fwd : GTTGCTACGAGAGGATTGTGTG Rev : ACCACTATAGCTGTGTCACCTG	AGAT	11	29	199-319	0.4
**19325**	Fwd : CTTCTTTAGCAAGGCAGTGGTC Rev : TGATATTAGTCTCTGGCCTGGC	AGAT	14	21	290-382	14.4
**333**	Fwd : GTTCCACTTCTCACGTGTACAC Rev : CGTGTATGAGCGACTATTCACC	ACAT	15	38	208-320	0.8

S2 Table

K	Reps	Model	Mean LnP(K)	Stdev LnP(K)	Ln'(K)	|Ln''(K)|	∆K
**1**	10	Ad	-7721.65	0.474342	NA	NA	NA
	10	AdLOCPRIOR	-7721.67	0.654981	NA	NA	NA
	10	NoAd	-7721.51	0.484080	NA	NA	NA
	10	NoAdLOCPRIOR	-7721.80	0.230940	NA	NA	NA
**2**	10	Ad	-7228.73	48.030385	492.92	16.65	0.346656
	10	AdLOCPRIOR	-7252.91	61.752741	468.76	2.54	0.041132
	10	NoAd	-7242.25	51.782027	479.26	28.91	0.558302
	**10**	**NoAdLOCPRIOR**	**-7232.92**	**38.846187**	**488.88**	**104.89**	**2.700136**
**3**	**10**	**Ad**	**-6752.46**	**4.293975**	**476.27**	**429.49**	**100.021539**
	**10**	**AdLOCPRIOR**	**-6781.61**	**9.185792**	**471.30**	**267.25**	**29.093844**
	**10**	**NoAd**	**-6791.90**	**60.826401**	**450.35**	**325.67**	**5.354090**
	10	NoAdLOCPRIOR	-6848.93	188.155320	383.99	237.75	1.263584
**4**	10	Ad	-6705.68	62.845150	46.78	191.41	3.045740
	10	AdLOCPRIOR	-6577.56	56.498480	204.05	186.49	3.300797
	10	NoAd	-6667.22	71.857170	124.68	27.11	0.377276
	10	NoAdLOCPRIOR	-6702.69	41.110973	146.24	64.06	1.558221
**5**	10	Ad	-6467.49	7.575905	238.19	177.50	23.429545
	10	AdLOCPRIOR	-6560.00	42.157245	17.56	15.20	0.360555
	10	NoAd	-6569.65	118.438951	97.57	41.34	0.349041
	10	NoAdLOCPRIOR	-6620.51	47.175240	82.18	62.43	1.323364
**6**	10	Ad	-6406.80	10.264610	60.69	36.96	3.600721
	10	AdLOCPRIOR	-6527.24	65.598157	32.76	45.66	0.696056
	10	NoAd	-6430.74	60.285252	138.91	72.89	1.209085
	10	NoAdLOCPRIOR	-6475.90	67.705129	144.61	78.69	1.162246
**7**	10	Ad	-6383.07	12.073299	23.73	NA	NA
	10	AdLOCPRIOR	-6540.14	75.539514	-12.90	NA	NA
	10	NoAd	-6364.72	56.543490	66.02	NA	NA
	10	NoAdLOCPRIOR	-6409.98	59.388772	65.92	NA	NA

S2 Fig

MODELS WITH HIGHEST VALUES OF ΔK

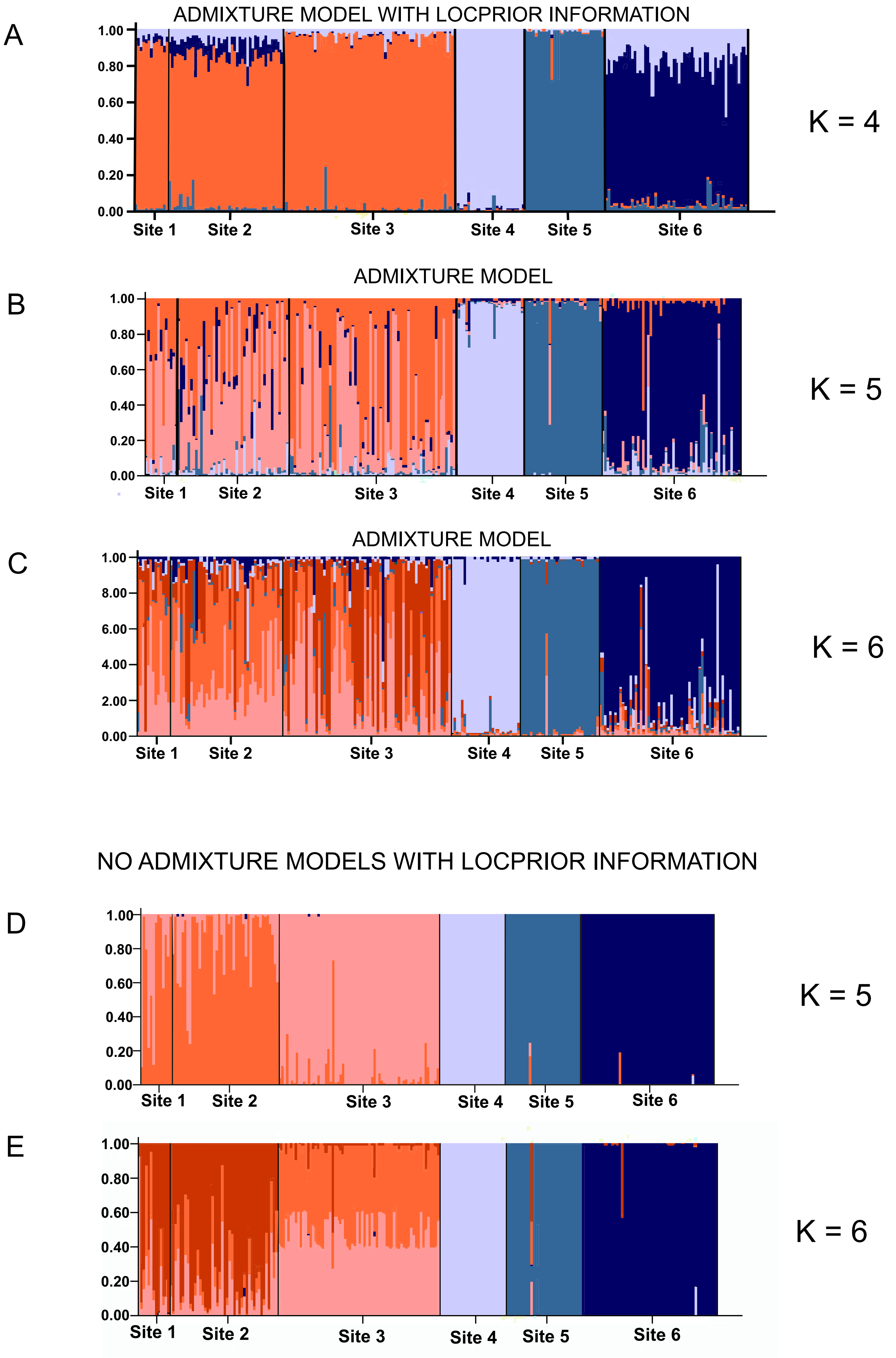

Supplement: S3 File — S1 Table gives the list of forward (Fwd) and reverse (Rev) primers for seven newly established microsatellite markers used for Mantidactylus bellyi. Repeat count are from the initial library. Percentage of missing data, numbers and length ranges of alleles refer to the entire dataset. Length range (inferred bp) includes primers and linker. S2 Table gives the inference of the population structure of M. bellyi based on Bayesian analysis for K = 1 to K = 7 for four models. Ad = Admixture model, AdLOCPRIOR = Admixture model with LOCPRIOR information, NoAd = No admixture model, NoAdLOCPRIOR = No admixture model with LOCPRIOR information. K = number of assumed subpopulations; Reps = number of MCMC iterations; ΔK = ad hoc statistic based on the change in the log probability data between successive K values. The most likely K is highlighted in bold. S2 Fig gives the genetic cluster assignment of the sampled populations of M. bellyi in Montagne d’Ambre. A–E] Individual assignments of M. bellyi to genetic clusters as inferred by STRUCTURE from a data set of seven microsatellites. A–C] The clustering scenarios of assumed subpopulations K = 4–6 were depicted for the models showing highest ΔK, i.e. A] Admixture model with LOCPRIOR information for K = 4, B] Admixture model for K = 5, and C] Admixture model for K = 6. D–E] The clustering scenarios were depicted for no admixture models with LOCPRIOR information of assumed subpopulations D] K = 5, and E] K = 6. (PDF) [file pone.0263764.s003.pdf]
